# Application of artificial intelligence approaches to predict the metabolism of xenobiotic molecules by human gut microbiome

**DOI:** 10.3389/fmicb.2023.1254073

**Published:** 2023-12-05

**Authors:** Aditya S. Malwe, Vineet K. Sharma

**Affiliations:** MetaBioSys Lab, Department of Biological Sciences, Indian Institute of Science Education and Research, Bhopal, India

**Keywords:** xenobiotic biotransformation, machine learning, artificial intelligence, human gut microbiome, drug designing

## Abstract

A highly complex, diverse, and dense community of more than 1,000 different gut bacterial species constitutes the human gut microbiome that harbours vast metabolic capabilities encoded by more than 300,000 bacterial enzymes to metabolise complex polysaccharides, orally administered drugs/xenobiotics, nutraceuticals, or prebiotics. One of the implications of gut microbiome mediated biotransformation is the metabolism of xenobiotics such as medicinal drugs, which lead to alteration in their pharmacological properties, loss of drug efficacy, bioavailability, may generate toxic byproducts and sometimes also help in conversion of a prodrug into its active metabolite. Given the diversity of gut microbiome and the complex interplay of the metabolic enzymes and their diverse substrates, the traditional experimental methods have limited ability to identify the gut bacterial species involved in such biotransformation, and to study the bacterial species-metabolite interactions in gut. In this scenario, computational approaches such as machine learning-based tools presents unprecedented opportunities and ability to predict the gut bacteria and enzymes that can potentially metabolise a candidate drug. Here, we have reviewed the need to identify the gut microbiome-based metabolism of xenobiotics and have provided comprehensive information on the available methods, tools, and databases to address it along with their scope and limitations.

## Introduction

Human gut microbiome (HGM) constitutes a community of more than 1,000 different bacterial species that colonise the human gut. HGM is majorly influenced by diet as well as various other factors like host genetics, geography, lifestyle habits, etc. As compared to the other body sites such as skin, scalp, oral and nasal cavities, and urogenital tract, human gut microbiome is highly diverse, dynamic with high density of bacteria belonging to various phyla like Bacteroidetes, Firmicutes, Actinobacteria, Proteobacteria and numerous other bacterial species. This community of gut bacteria contribute more than 3.3 million unique genes that provide the human host with several folds higher metabolic capabilities than contributed by its own genome (Guinane and Cotter, 2013; Jethwani and Grover, 2019).

Gut microbiota derived secondary metabolites like vitamin B12, thiamine, riboflavin, biotin etc. have beneficial effects on the host (Gomaa, 2020). The commensal gut bacteria can metabolise indigestible dietary fibres and other dietary bioactive molecules such as polyphenols, oligosaccharides, pigments, etc. to produce important secondary metabolites such as short chain fatty acids like butyric acid and propionic acid that have anti-carcinogenic, anti-inflammatory properties as well as act as energy source for intestinal cells (Cencic and Chingwaru, 2010). As a result, HGM have crucial effect on human health and physiology through their multifaceted influence on metabolic and immunological systems. On the other hand, dysbiosis or alteration in abundance of commensal gut bacteria can lead to various inflammatory disorders like IBD, Crohn’s disease, colon cancer and metabolic disorders like obesity and diabetes (Blandino et al., 2016). As a result, several recent studies are now focusing on exploiting the metabolic capabilities of gut microbiome as therapeutical target to modulate human health (Brandt, 2013; Suez et al., 2018; Sokol et al., 2020).

The extensive metabolic capabilities also allow gut bacteria to metabolise orally administered drugs and other pharmaceutical and nutraceutical xenobiotics. This direct gut bacterial biotransformation of drugs and other xenobiotics are known to alter the pharmacological properties and intended effect on the human host, as well as cause loss of bioavailability due to depletion (Ilett et al., 1990; Wilson and Nicholson, 2017). During drug development stages, susceptibility of the drugs to gut microbial metabolism is often overlooked (Wilson and Nicholson, 2017). This results in unresponsiveness to drug therapy, unintended toxic effects and in dose formulation due to microbial depletion of drugs.

For example, digoxin, a cardiac glycoside is reduced to its inactive form dihydrodigoxin by *Eggerthella lenta* (Haiser et al., 2013). Similarly, gut bacteria mediated biotransformation can also lead to increased toxicity of certain drugs like irinotecan (Brandi et al., 2006). This issue is further exacerbated by enzyme promiscuity, where certain enzymes can metabolise substrates that are structurally similar to their natural substrate (Khersonsky et al., 2006). In fact, the biotransformation of digoxin is an example of enzyme promiscuity. Fumarate is the natural substrate for Cgr reductase present in *Eggerthella lenta*. However, it can also metabolise digoxin due to the partial structural similarity between digoxin and fumarate (Haiser et al., 2014).

Gut bacteria can perform various kinds of biotransformation such as reduction, demethylation, hydrolysis, deamination etc. that can potentially alter the efficacy of xenobiotics (Wilson and Nicholson, 2017). Traditionally, Nuclear Magnetic Resonance (NMR) and Liquid Chromatography-Mass Spectrometry (LC–MS) are used for metabolic profiling followed by metagenomic and microbiological experiments for identification of gut bacterial species involved in such biotransformation. However, owing to the complex and dynamic gut microbial interactions and vast array of metabolic enzymes that can biotransform xenobiotics, employing such experimental methods create a bottleneck for studying bacterial species-metabolite interactions in gut. Thus, computational approaches and machine learning-based tools are now preferred to predict gut bacteria that can potentially metabolise a candidate drug. In this review, we highlight the complexity and mechanistic insights into the different computational approaches to predict the biotransformation of xenobiotic molecules by human gut microbiome while focusing more on artificial intelligence (AI) and machine learning (ML)-based approaches, and also discussing the existing resources and databases that can be used to develop new prediction tools.

## Gut microbiome mediated metabolic reactions

Typically, there are three common and key components of any xenobiotic biotransformation in the human gut. One component is the enzyme, i.e., the gut bacterial enzyme that can catalyse the said biotransformation or the reaction. Another is the gut bacterial species harbouring the metabolic enzyme, and the third one is any orally ingested biotic or xenobiotic molecules that could be a potential substrate for the gut bacterial enzyme.

**Figure 1:**
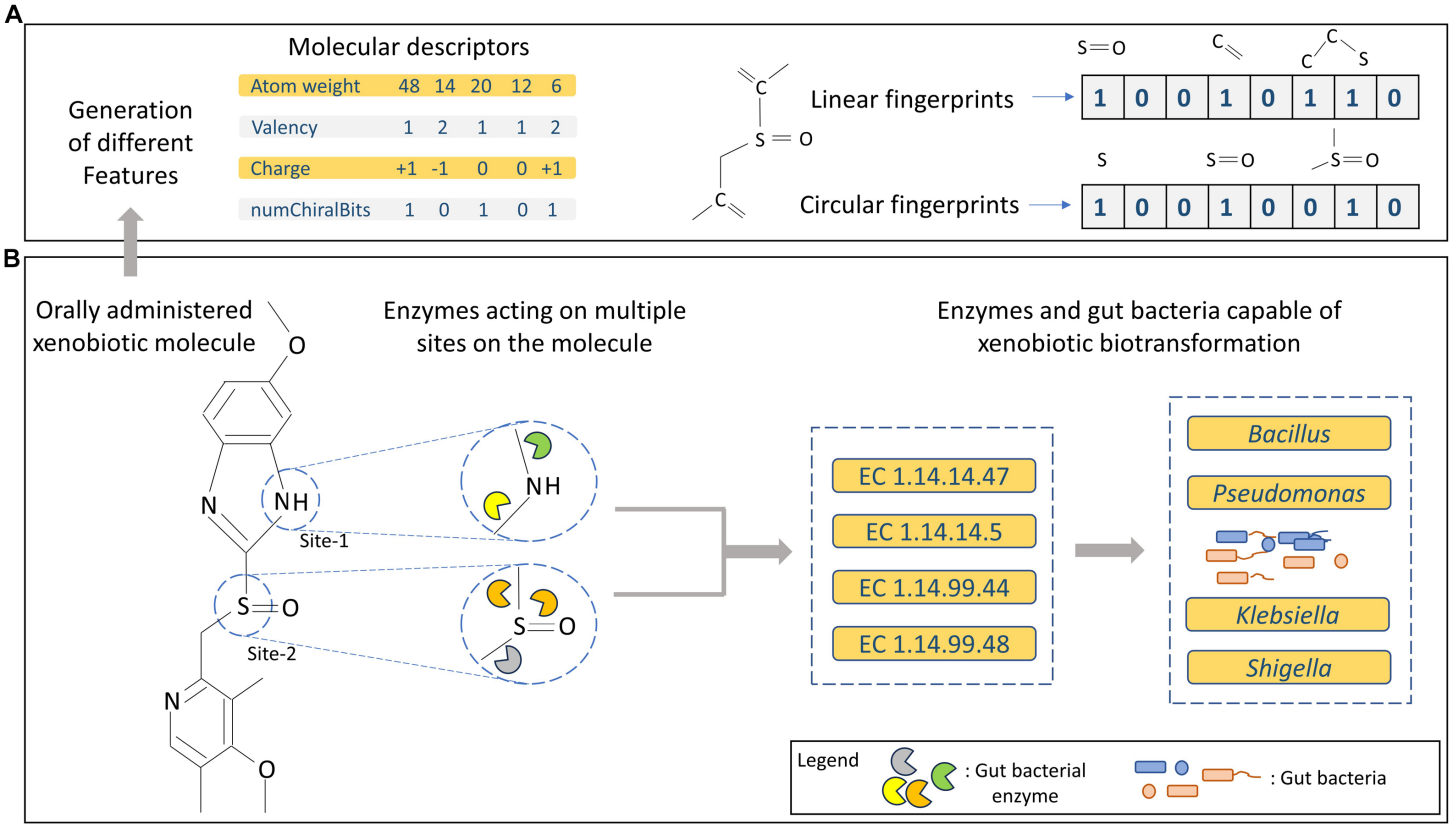
**(A)** Representation of different types of features generated for molecular/substrate data. **(B)** Overview of multi-site metabolism of xenobiotics along with enzymes and gut bacteria harbouring them.

At the structural level, biotic and xenobiotic molecules possess multiple functional groups or structural moieties that can serve as potential sites for enzymatic actions (Hult and Berglund, 2007; Li et al., 2011). Thus, a major fraction of all existing orally administered drugs/xenobiotics may be subjected to gut bacterial metabolism. However, the presence or absence of certain functional groups could make them more prone to biotransformation (Zimmermann et al., 2019; Javdan et al., 2020). As a result, a single substrate can be metabolised by different types of enzymes leading to a variety of products arising from a single substrate (Figure 1B). Depending on the type of enzyme involved in the biochemical process, different reactions can occur through variety of mechanisms, thus enzymes are categorized into different groups based on the type of reaction they catalyse, as discussed in the following text.

## Different classes of metabolic enzymes

Enzymes are biochemical agents that catalyse the conversion of a biochemical substrate into a product. Enzymes are broadly classified into six reaction classes system devised by International Union of Biochemistry and Molecular Biology (IUBMB) i.e., 1. Oxidoreductase, 2. Transferase, 3. Hydrolase, 4. Lyase, 5. Isomerase, and 6. Ligase. They are classified by a unique Enzyme Commission number (EC number) containing four digits that identifies the complete reaction performed by an enzyme. The first digit of the EC number identifies the reaction class of an enzyme (type of reaction), the second digit identifies reaction subclass within the reaction class of an enzyme (type of substrate or nature of functional group), the third digit identifies the reaction sub-subclass within the reaction subclass, and the fourth digit identifies the name of the substrate (Cornish-Bowden, 2014). The gut microbiome harbours a diversity of metabolic enzymes, which are primarily dominated by EC1 and EC2 class of enzymes, and the least abundant enzyme classes are EC5 and EC6.

## Metabolic complexity of human gut microbiome

Taken together, the presence of more than 4,500 gut bacterial species harbouring more than 350,000 metabolic enzymes from the six different enzymatic classes provides an enormous functional diversity in HGM to biotransform a vast and diverse array of xenobiotics through various mechanisms (Almeida et al., 2021). The most prevalent enzymatic biotransformations by gut bacteria are carried out by EC1 (oxidoreductase) class, followed by EC2 (Transferase) and EC3 (Hydrolase) (Jourova et al., 2016) that aligns well with the fact that the gut environment provides optimum conditions for reductive reactions (Guo et al., 2020).

Though the reduction reactions are the most common type of reactions carried out by gut bacteria, various other reactions across different reaction classes such as deacylation, demethylation, hydrolysis, dehydroxylation etc. are also involved in the biotransformation of xenobiotics (Wilson and Nicholson, 2017; Guo et al., 2020).

## Gut microbial cross-feeding interactions

**Figure 2:**
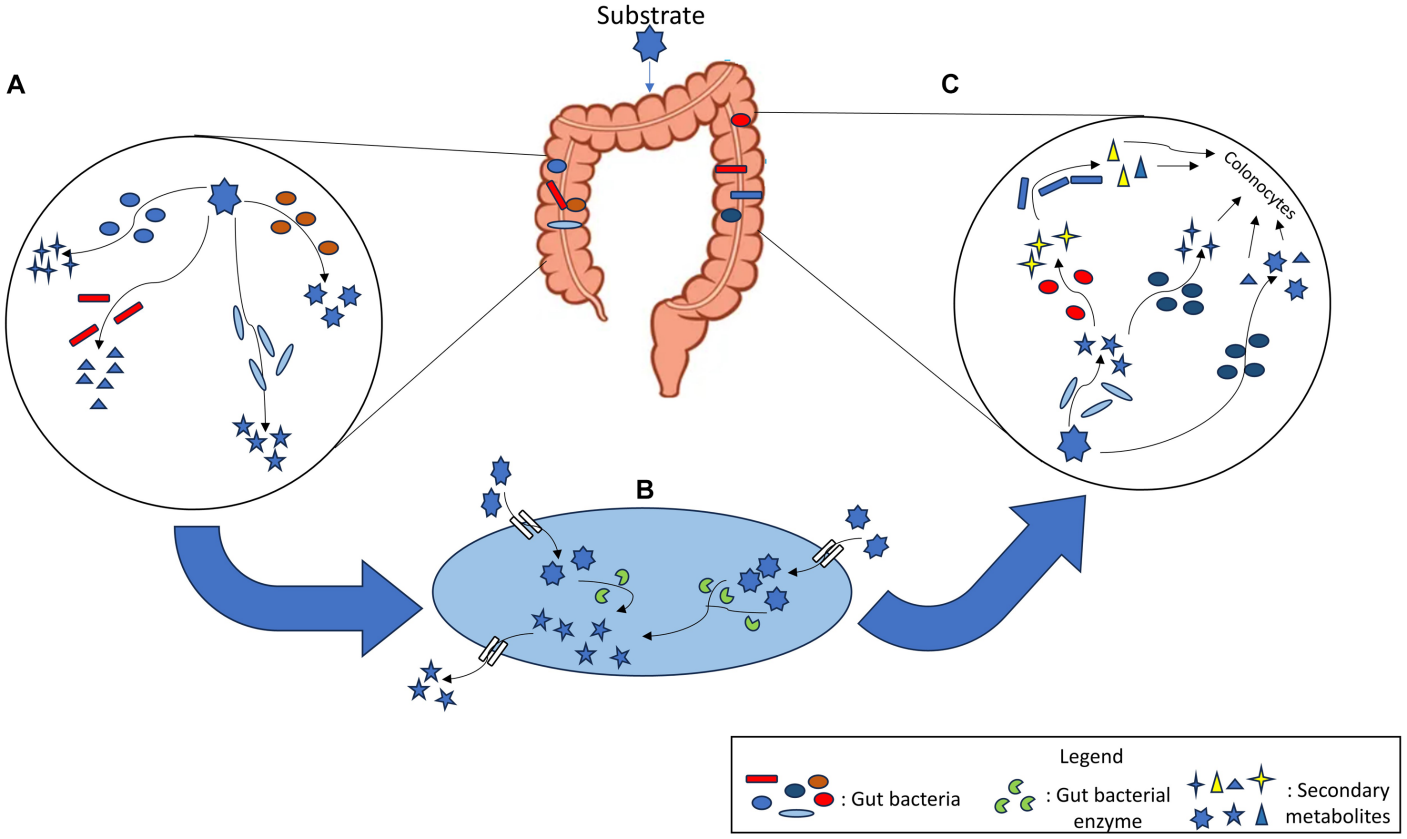
**(A)** Single substrate can be metabolised by multiple gut bacterial species into different products. **(B)** Within a bacterial cell, enzymes act on the xenobiotic substrate which is metabolised and converted to another metabolite. This metabolite is released into the gut lumen and is available for other bacteria. **(C)** Secondary metabolites generated after biotransformation of xenobiotic are used by other gut bacterial species that again metabolise them into new products. Thus, a cascade of reactions occurs, the products of which are made available for other members of gut microbial community as well as colonocytes.

Another level of metabolic complexity in human gut emerges from the positive and negative interactions of microbial communities via sharing of metabolites among them. This phenomenon, referred as metabolite cross-feeding helps in funnelling nutrients and metabolites across the community as well as to the host (Culp and Goodman, 2023). This metabolite cross-feeding also applies to xenobiotic biotransformation via which a metabolite may undergo a cascade of biochemical reactions where the product from a given reaction can be used as a substrate for the next set of reaction by various gut bacteria. As a result, xenobiotic biotransformation often leads to pharmacological alterations of drugs efficacy as well as affects gut bacterial communities and host health by selecting gut bacteria capable of biotransforming xenobiotics such as complex prebiotics or nutraceuticals. Further to the above complexity, a given molecule contains multiple functional groups that may serve as multiple sites for diverse enzyme catalysed biochemical conversions. Thus, a single molecule can undergo biotransformation through diverse reaction mechanisms (Figure 2). Enzyme promiscuity further amplifies the metabolic capabilities of gut microbiome to perform the unintended metabolism of xenobiotics such as the cases of drugs like digoxin, levodopa (Zhu et al., 2016), gemcitabine (Geller et al., 2017) and sulfasalazine (Collins and Patterson, 2020).

## Enzyme-substrate interactions: specificity and promiscuity

It is important to understand and examine how an enzyme binds to a substrate during the catalytic conversion to their corresponding biological products for which various models have been proposed. The lock-and-key model (Eschenmoser, 1995) was one of the earliest models that proposed strict and rigid structural specificity between enzyme and substrate. This notion was challenged by Koshland’s induced fit hypothesis (Koshland, 2004) postulating that enzyme structure is flexible and specific substrates can induce structural changes in enzyme structure thus enabling optimal enzyme-substrate interactions leading to catalysis (Babtie et al., 2010). The interaction between functional groups of the substrates and those found in the active site of enzyme are presumed to be optimised for a specific biochemical mechanism based on the stereochemistry of substrate-enzyme complex.

However, molecules with a structure similar to the native substrate can also occupy the active site of that enzyme leading to biotransformation of the non-native substrate. This phenomenon is known as enzyme promiscuity (Khersonsky et al., 2006; Babtie et al., 2010). There are several known examples of enzyme promiscuity where the mechanism of biotransformation of a native and a non-native substrate is similar. A widely studied example of enzyme promiscuity in gut bacterial biotransformation of xenobiotics is that of digoxin (Haiser et al., 2013). Digoxin is a cardiac glycoside consisting of glycon (composed of carbohydrate) and aglycon (composed of steroid) moieties. Cgr reductase, also known as fumarate reductase present in gut bacterial species *Eggerthella lenta* can reduce the lactone ring present in digoxin’s aglycon moiety, thus biotransforming digoxin into its therapeutically inactive form dihydrodigoxin (Haiser et al., 2014). A part of digoxin drug structure is similar to fumarate, which is the native substrate of Cgr reductase. Thus, digoxin is capable of occupying the active site of Cgr reductase with a lower affinity leading to the reduction of digoxin’s lactone ring (Kumar et al., 2018). Besides digoxin, almost 150 drugs are recently found to be influenced by gut bacterial xenobiotic metabolism due to promiscuous enzyme activities (Zimmermann et al., 2019). Such metabolisms can lead to various unintended effects such as increased toxicity, loss of drug efficiency as well as increased efficiency in some cases.

Other examples such as inulin or lactulose are prominent nutraceuticals prescribed as prebiotics that undergo metabolism by phosphorylase and hydrolases present in *Bifidobacterium* and *Lactobacillus* and promotes the growth of these probiotic species (Roberfroid et al., 1998; Thompson, 2002). Thus, the prediction of the exact metabolic reaction for a candidate prebiotic or nutraceutical substrate, the enzyme carrying out the reaction and the bacteria harbouring it, is crucial to identify and develop efficient prebiotics/nutraceuticals and probiotics.

## Identification of promiscuous substrates by molecular similarity

In an enzyme catalysed promiscuous reaction, a molecule with close structural similarity to the native substrate of an enzyme can fit into the active site of enzyme and undergo biotransformation. Thus, it is desired to identify and cluster the biomolecules that are structurally similar to enzyme substrates to predict the promiscuous metabolic reactions. Various structural similarity metrics such as Tanimoto similarity coefficient (Bajusz et al., 2015), cosine coefficient, Euclidean distance, substructure and superstructure similarity search (Yan et al., 2005; Klinger and Austin, 2006) etc. can be used to calculate the structural similarity between two molecules. Of these, Tanimoto similarity coefficient is a widely used metric to measure the similarity between molecules, and is defined as ratio of intersection between two sets of elements in molecules and can be calculated as follows:


$$TC=\frac{z}{x+y-z\text{.}}$$

where, *x* is the number of bits obtained from fingerprints of molecule A, *y* is the number of bits obtained from fingerprints of molecule B, and *z* is the union of bits obtained from fingerprints of both molecules A and B.

Using structural similarity coefficients, the degree of overlap between two molecules or between a molecule and the native substrate of an enzyme can be determined. A higher value of Tanimoto coefficient equates to a higher degree of structural resemblance between a molecule and native substrate and increases the possibility of a promiscuous metabolism. In some recent works, a value of 0.8 has been used as the threshold value to determine optimal structural overlap between two molecules (Jaiswal et al., 2021).

## Population and individual specific xenobiotic biotransformation

Another key factor in gut microbiome mediated xenobiotic metabolism arises from the noteworthy differences in the gut microbiome compositions of different populations due to the differences in the diets, lifestyles and geographical locations. As a result, the possible biotransformation of a given xenobiotics may vary between different populations. The earlier cited example of the promiscuous metabolism of Digoxin is also an example of population-specific metabolism, where the prevalence of digoxin biotransformation was significantly higher in American patients as compared to healthy south Indian and Bangladeshi patients (Alam et al., 1988; Mathan et al., 1989). Thus environment, lifestyle, dietary habits, and other factors that shape the gut microbiome in different populations also defines the gut-microbiome mediated metabolic capabilities and the resultant differences in gut microbe-mediated xenobiotic biotransformation. This also applies to the gut microbiome variations between different individuals belonging to the same or a different population. Such examples highlight that xenobiotic biotransformation, and its subsequent effects can vary from person to person and more individual and population centric approaches for studying gut microbe-mediated biotransformation are required.

## Consequences of xenobiotic biotransformation

To summarize the above, it is apparent that human gut bacteria can utilise different types of mechanisms to biotransform structurally diverse xenobiotics to alter their pharmacological properties. These alterations can lead to either inactivation of drugs, conversion of prodrug into its active form or increased toxicity of metabolised drugs. Nucleoside analogue Gemcitabine (2′,2′-difluorodeoxycytidine) is used as chemotherapeutic drug for the treatment of different types of cancers including aggressive forms of cancer like pancreatic ductal adenocarcinoma (PDA). Various studies have linked gemcitabine inactivation by bacteria colonising tumour tissues such as *Mycoplasma hyorhinis* (Vande Voorde et al., 2015; Geller et al., 2017). Deamination of gemcitabine by bacterial cytidine deaminase converts gemcitabine into its inactive form 2′,2′-difluorodeoxyuridine (dFdU) that is excreted out, leading to ineffective antitumour response (Abbruzzese et al., 1991; Frese et al., 2012). Another example is the case of digoxin that gets inactivated by the reduction of its lactone ring and convert to dihydrodigoxin by Cgr reductase in *Eggerthella lenta* species (Haiser et al., 2013).

Likewise, gut microbial biotransformation can also increase the efficacy of drugs by converting a prodrug into its active form. An example is Sulfasalazine, which is prescribed as an anti-inflammatory prodrug for the treatment of ulcerative colitis (Klotz et al., 1980). Sulfasalazine contains two structural moieties, 5-aminosalicylic acid (5-ASA) and sulfapyridine that are coupled together with diazo linkage. Azoreduction performed by gut bacterial azoreductase cleaves the diazo coupling to release 5-ASA that has anti-inflammatory activity, thus reducing pro-inflammatory conditions associated with ulcerative colitis (Carmody and Turnbaugh, 2014; Crouwel et al., 2021). Similarly, gut bacteria mediated hydrolysis converts lovastatin into its active β-hydroxy acid form (Yoo et al., 2014; Enright et al., 2016).

In a few cases, gut bacterial biotransformation is also found to enhance the toxicity of prodrugs such as irinotecan. Prodrug irinotecan is a chemotherapeutic drug that is administered for treatment of colorectal cancer. Irinotecan (SN-38) is cytotoxic and prior to its entry into intestine, hepatic UDP-glucuronosyltransferases convert SN-38 into its glucuronidated nontoxic form SN-38G. However, β-glucuronidases found in various bacterial genera like *Clostirdium, Faecalibacterium, Bacteroides* etc. reconverts SN-38G into its cytotoxic form SN-38 that can cause adverse drug response and severe diarrhoea (Guthrie et al., 2017; Collins and Patterson, 2020). Various tools such as ToxiM and admetSAR are available for toxicity prediction of a molecule (Cheng et al., 2012; Sharma et al., 2017b). Toxicity prediction tools predict various properties of a molecule such as their solubility, absorption, excretion and, metabolism by host, and also predict any direct toxic effect on the host (Hutchinson et al., 1979; van Breemen and Li, 2005). ToxiM and admetSAR utilise machine learning-based classification and regression models to predict toxicity of input molecules (and biomolecules in case of admetSAR). Toxicity prediction is an important aspect of understanding gut bacteria mediated xenobiotic metabolism as toxic byproducts of unintended gut bacterial metabolism may lead to severe health complications.

## Bioaccumulation of xenobiotics as novel mechanism of drug depletion

Recent studies have highlighted bioaccumulation of drugs as another mechanism adopted by gut bacteria that can alter availability of drugs/xenobiotics (Cohen and Kelly, 2022). Bioaccumulation has a direct impact on drug bioavailability as accumulation of drug molecules inside gut bacterial cells depletes the drug concentration that in turn attenuates drug response in the host. In addition, bioaccumulation affects bacterial cell physiology and induces metabolic cross-feeding interactions with other bacteria from the gut bacterial community. Many xenobiotics such as duloxetine, montelukast, roflumilast etc., are accumulated inside bacterial cells without undergoing biotransformation (Klünemann et al., 2021). Metabolic enzymes are involved in this bioaccumulation where such enzymes bind to a drug molecule without any biotransformation and leads to its unavailability. For example, duloxetine can bind to nucleotide biosynthesis pathway enzymes without undergoing biotransformation in *Clostridium saccharolyticum* (Klünemann et al., 2021). More experimental evidence is required to obtain deeper insights into the process and impact of bioaccumulation on host as well as to identify the kind of drugs that could be susceptible to bioaccumulation.

## Determining xenobiotic metabolism by experimental methods

The experimental determination of enzymatic biotransformation of a given xenobiotic by a gut bacterium is a challenging task (Koppel et al., 2017). To identify the biotransformation/bioaccumulation of xenobiotics by human gut microbiome, expensive techniques like nuclear magnetic resonance, liquid chromatography-mass spectrometry are required along with a library of xenobiotic molecules to screen for possible metabolism (Zimmermann et al., 2019; Javdan et al., 2020). Further, to identify the gut bacteria involved in this biotransformation and the mechanism of biotransformation, culture-based analysis and biochemical assays are required which are tedious tasks. Furthermore, the dynamic nature of human gut microbiome and complexities associated with gut microbe-mediated metabolisms makes experimental determination of a xenobiotic biotransformation a tough task. In this scenario when experimental methods have their own limitations, the various computational approaches such as docking, molecular dynamics simulations, development of artificial intelligence-based computational tools/resources to predict the potential candidate molecules that can undergo gut bacteria mediated biotransformation are helpful to narrow down the number of molecules to be tested experimentally. Such computational tools can thus help in reducing the cost and time for experimental validations of potential biotransformation of a given candidate molecule.

## Computational approaches for studying gut bacterial xenobiotic biotransformation

Biochemical processes such as xenobiotic biotransformation involves enzyme-substrate interactions for which the computational methods such as molecular dynamics simulations and molecular docking have been widely used. Molecular docking helps to study the binding, affinity and optimal conformation for enzyme-substrate complex by simulating molecular interactions between substrate and active site of the enzyme (Morris and Lim-Wilby, 2008). Molecular dynamics simulations study wider details such as protein folding, conformational changes, ligand binding, etc. (Hollingsworth and Dror, 2018). Both processes have been extensively used to identify potential ligands and their target enzymes. A large repertoire of software are also available that have implemented docking and molecular dynamics simulations for this purpose (Chen et al., 2003; Verdonk et al., 2003; Lang et al., 2009) Molecular dynamics simulation was successfully applied to study the binding of digoxin and amphetamine into the active site pocket of Cgr reductase and tyramine oxidase enzymes, respectively (Kumar et al., 2018, 2019). However molecular docking and molecular dynamics simulations have limitations due to the lack of availability of complete 3D structure for many enzymes, thus restraining their application towards deciphering novel substrate-enzyme interactions (Fan et al., 2019).

Besides molecular dynamics simulations and docking, Quantitative structure–activity relationship (QSAR) modelling is also widely used to predict the bioactivity of drugs and xenobiotics based on their structure (Cherkasov et al., 2014). QSAR methods identify molecules with known properties that are structurally similar to a query molecule and extrapolate their properties to the query molecule. However, since QSAR models study individual molecules, they have limited ability to predict biotransformation (Cherkasov et al., 2014).

The application of AI/ML-based approaches are gaining significance due to their ability to predict the possible enzymatic reactions and enzyme-substrate interactions. AI/ML-based methods involve statistical methods to understand and learn from a dataset and make predictions. Usually, supervised learning is implemented that uses labelled data and makes predictions as per the available labels. Multiple ML-based methods such as random forest (RF) (Ali et al., 2012), support vector machine (SVM) (Suthaharan, 2016), artificial neural networks (ANN) (Krogh, 2008) etc. are available that can perform classification as well as regression tasks. Before moving to the section “Artificial intelligence and machine learning for predicting xenobiotic biotransformation,” which details the methodology used in AI/ML-based prediction of xenobiotic metabolism, it is required to understand the training datasets and their sources which is discussed in the following section.

## Databases for gut microbes, compounds, and substrates information

The application of machine learning or AI-based approaches require curated and adequate information for comprehensive training for making reliable predictions. For any microbiome-based biotransformation of a given molecule, the key input needed are the enzymatic reactions in various EC classes, substrate, product, and the bacteria that harbour the metabolizing enzymes. The following section provides an overview of the available resources to construct a comprehensive training dataset.

Various comprehensive databases exist that contain computer readable molecular information for diverse array of compounds. PubChem (Kim et al., 2023) is an open chemistry database maintained by National Institute of Health (NIH) that contains chemical information on chemical structure, physical properties and biological activities of vast array of small and large molecules. For each molecule, chemical information is available in various formats like .sdf, .mol, .smi, etc. In addition to PubChem, other databases such as KEGG (Kyoto Encyclopaedia of Genes and Genomes) (Kanehisa, 2000), UniProt (UniProt Consortium, 2015) and BRENDA (BRaunschweig ENzyme DAtabase) (Scheer et al., 2011) contain complete metabolic information for all enzyme reactions, including the information regarding substrates, products, reaction type and EC number of enzymes involved in the reaction.

KEGG is a collective database for linking genomic information with metabolic and functional information on cellular processes, enzymes, and enzyme-substrate pairs (Kanehisa, 2000). KEGG also provides information on various metabolic pathways along with detailed information regarding the participating substrates, cofactors and resultant products. KEGG also provides ortholog database that links molecular functions with different genes and metabolic pathways thus creating functional ortholog database. KEGG orthologs can be used for constructing reference databases for prediction of metabolic profiles using microbiome abundance data as used in Mangosteen (Yin et al., 2020) and MIMOSA (Noecker et al., 2016). For example, KEGG ortholog K00161 provides information about pyruvate dehydrogenase E1 subunit that includes it EC number (EC 1.2.4.1), its reaction involving pyruvate and thiamine diphosphate as substrate and 2-alphahydroxyethyl thiamine as its product, along with the pathways in which this enzyme participates. Such information can be used to retrieve the substrates, products and the corresponding reactions, which can be used for training the prediction models. BRENDA is another curated database that provides information on functionality, structure, and occurrence of enzymes across biological systems (Scheer et al., 2011). Together both these databases include comprehensive information on metabolism, pathways and ligands involved in biochemical processes.

Using such databases, information regarding enzymes that can metabolise xenobiotics can be obtained. Further, the databases containing information on gut bacteria such as Virtual Metabolic Human (VMH) (Noronha et al., 2019) provide complete information regarding gut bacterial strains colonising the human gut. Human Microbiome Project (HMP) also provide consolidated gut microbial database as well as raw metagenome reads, along with bioinformatic tools for analysis (Turnbaugh et al., 2007; Gevers et al., 2012). Besides these databases, NCBI genome browser also maintains genbank and refseq sequence databases to provide genomic information for various gut bacterial species and strains (Pruitt, 2004). Thus, the information on enzymes that can metabolise xenobiotics can be linked to the gut bacterial abundance data to estimate the role of different bacterial species in different populations in the metabolism of a given xenobiotic (Table 1).

**Table 1:** Information regarding different types of databases useful for studying gut bacteria mediated xenobiotic biotransformation.

Databases	EC number information	Protein sequences	Biochemical pathways	Molecular data files (.sdf, .mol,.smi)	Gut bacterial abundance profiles
BRENDA	✔	✔	**×**	**×**	**×**
KEGG	✔	✔	✔	✔	**×**
Expasy	✔	✔	✔	**×**	**×**
Pubchem	**×**	**×**	**×**	✔	**×**
HMP	**×**	✔	**×**	**×**	✔
VMH	**×**	**×**	**×**	**×**	✔

KEGG, Kyoto Encyclopaedia of genes and genomes; HMP, human microbiome project; VMH, virtual metabolic human.

Using nucleotide and protein sequences from these databases, taxonomic identification of gut bacterial species and their functional annotations can be obtained. Bracken (Lu et al., 2017) provides species level bacterial abundance from metagenomic datasets. MetaPhlAn2 (Truong et al., 2015) is another tool that is useful for accurate reconstruction of taxonomic composition from shotgun metagenome sequences. MetaBin (Sharma et al., 2012) uses blat for taxonomic assignment of short reads whereas 16S classifier (Chaudhary et al., 2015) is a machine learning-based tool that uses random forest for taxonomic identification of bacteria using short hypervariable regions of 16S rRNA gene sequence. Similarly, tools such as eggnog-mapper (Cantalapiedra et al., 2021), Woods classifier (Sharma et al., 2015) and MetaBioME (Sharma et al., 2010) can be used for functional annotation and identification of enzymes from different metagenomic datasets.

## Artificial intelligence and machine learning-based approaches

Machine learning can be broadly defined as the computational process of constructing prediction models using statistical methods on the correlated informative groups within the data to predict properties of new data points (Tarca et al., 2007; Greener et al., 2022). Machine learning-based prediction tools can be trained for classification (predicting discrete categories), regression (predicting continuous values) or clustering (predicting groups or clusters within the data) problems.

Based on the type of problem, machine learning can be broadly classified into supervised learning and unsupervised learning. In supervised learning, the training is carried out using labelled data, and each data point is annotated with at least one label that represents the category to which the data point belongs. The objective of supervised learning is to learn patterns within the labelled data and predict a label for any new input data (Nasteski, 2017). Supervised machine learning deals with classification and regression problems. On the other hand, unsupervised machine learning utilises unlabelled data for training, and identifies patterns within the data, and groups the data points into different clusters (Usama et al., 2019), making it suitable for clustering problems.

For supervised machine learning, different types of methods such as decision trees, support vector machine, neural networks are widely used. Decision tree (Batra and Agrawal, 2018) involves utilizing tree-like structure for predicting outcome for any input data. The structure of decision tree consists of nodes and edges, with each feature representing nodes of the tree. Based on the threshold function of input feature, the edges spread into the feature space incorporating new features (Tarca et al., 2007). Random forest is a powerful classifier that involves ensemble of decision trees where collective vote of all the trees in the forest becomes the final prediction output of random forest (Ali et al., 2012). Support vector machine is another powerful algorithm that creates hyperplanes that mark the plane of separation and prediction across different labels (Suthaharan, 2016). Hyperplane used in SVM is the plane of separation of different types of classes present in the high-dimensional dataset (Noble, 2006). This hyperplane of separation is selected by maximising margin between the hyperplane and the nearest data points and also by utilising kernel functions that creates hyperplane at higher dimensions for good separation between different classes. SVM can be used for classification as well as regression problems. Artificial neural network is a highly versatile and customisable algorithm applicable for binary, multiclass as well as multilabel classification problems. ANN architecture involves nodes arranged in multiple layers where computation, processing and analysis is performed (Krogh, 2008).

All machine learning approaches aim to learn from the underlying relationship between various features of the dataset while maximising the ability of the models to be generalized, i.e., to be able to give correct predictions on input data that is not used during training. The phenomenon where a machine learning model fails to learn from the available features is termed as “underfitting.” Such models fail to provide accurate predictions as a result of lack of adequate training. Similarly, if a model starts learning from the noise present in the training data due to overtraining, it results in “overfitting.” Such models perform best on the training data but fail to replicate their performance on any data other than the training data. Proportion of positive and negative class in the training data can also affect performance of the models, and is expected to be nearly equal proportions for optimal training. Underfitting can be solved by diversifying the dataset used for training and increasing the size of the dataset. Overfitting can be solved by simplifying the model by reducing the number of parameters used during training (Greener et al., 2022). Addressing the issues of underfitting and overfitting improves the accuracy and overall performance of any machine learning models. Interestingly, certain methods such as random forest are intrinsically resistant to problem of overfitting due to internal validation measures such as Out-of-bag error estimation (Cutler et al., 2012). Method called bootstrapping that uses random sampling of data is used to separate a set of random samples from the complete dataset during construction of trees in random forest. The unsampled data is referred to as out-of-bag data which is used to validate the performance of random forest after its training on bootstrapped data. This makes random forest resistant to overfitting.

## Binary, multiclass and multilabel classification

Depending on the number of classes to be predicted, machine learning classifiers are termed as binary, multiclass or multilabel classifiers. Binary classifiers are simplest form of classifiers involving prediction between two mutually exclusive classes, for example to predict whether a protein sequence will have proinflammatory effect on immune system or not (Gupta et al., 2016). In multiclass classification, more than two mutually exclusive classes are present, for example predicting multiple types of cells from a cell population seen in a microscopy image (Misselwitz et al., 2010). In both the above cases, a single data point exclusively belongs to a single class. On the other hand, in case of multilabel classification a single data point can belong to multiple classes, for example a single molecule can be metabolised by enzymes from multiple EC classes (Scheer et al., 2011).

Random forest is a powerful algorithm that can be used for binary, multiclass or multilabel problems whereas SVM is suitable for binary and multiclass classification problems but not for multilabel problems. Other algorithms such as ANN, Naïve Bayesian (Yang, 2018) and K-nearest neighbours (KNN) (Taunk et al., 2019) can also be directly used for binary and multiclass classification. However, for proper implementation of multilabel classification, two broad categories of methods are available; problem transformation and algorithm adaptation (Pushpa and Karpagavalli, 2017). Problem transformation methods convert the multilabel classification problem into simpler binary or multiclass problems by transforming the data labels using various methods such as binary relevance, label power set, classifier chain etc. are available (Zhang and Zhou, 2014). On the other hand, algorithm adaptation involves utilising traditional machine learning methods that are modified to deal with multilabel classification such as ML-kNN (Zhang and Zhou, 2007), ML-DT (Clare and King, 2001), Rank-SVM (Elisseeff and Weston, 2002) etc.

## Evaluation metrics for different types of classification problems

The performance of various machine learning-based methods can be evaluated using different metrics such as accuracy, precision, recall, F1-score, hamming loss etc. that highlight different aspects of the model. Based on the predictions obtained on test set or validation set, confusion matrix consisting of true positive (TP), true negative (TN), false positive (FP) and false negative (FN) values is constructed. Using these values, different metrics are calculated.

Accuracy is defined as proportion of correctly predicted instances out of all instances and is calculated as


$$\mathrm{Accuracy}=\frac{TP+TN}{TP+TN+FP+FN}$$

Precision is defined as ability of the classifier to differentiate between positive and negative instances in the dataset, i.e., out of all positive predictions, how many were actually positive and not false positive. Precision is calculated as


$$\mathrm{Precision}=\frac{TP}{TP+FP}$$

Recall defines the ability of classifier to correctly predict true positive instances from the dataset and is calculated as


$$\mathrm{Recall}=\frac{TP}{TP+FN}$$

Using precision and recall, another key metric called F1 score can be calculated that measures the proportion of correct predictions from all the positive predictions. F1-score is harmonic mean of precision and recall and is calculated as


$$F1-\mathrm{score}=2x\;\frac{\mathrm{Recall}\;x\;\mathrm{Precision}}{\mathrm{Recall}+\mathrm{Precision}}$$

The above-mentioned metrics are routinely used for binary as well as multiclass classification problems for evaluating different machine learning methods and models (Dalianis, 2018). However, due to complexities associated with multilabel classification and problem transformation, accuracy alone is not the appropriate metric to assess the performance of classification models (Tsoumakas et al., 2006). Hamming loss (Wu and Zhu, 2020) and binary accuracy are also among the widely used metrics for evaluating performance of multilabel classification models (Jaiswal et al., 2021). Hamming loss is defined as fraction of incorrectly predicted labels from the complete set of available labels in the dataset. Hamming loss thus represents the error rate of the multilabel classification models and should be as low as possible. Binary accuracy is the accuracy of a single label from the complete set of available labels and is calculated for all labels independently. Thus, based on the classification problem under focus, appropriate evaluation metrics should be selected to assess the performance of the prediction models. To evaluate the performance of machine learning models, validation is performed on data (validation set) that was not used during the training. Thus, the validation set helps in assessing the performance of prediction models on real data and also helps in identifying any bias during training of the models.

## Artificial intelligence and machine learning for predicting xenobiotic biotransformation

Machine learning-based methods can be used for predicting gut bacterial enzymes and complete biochemical reactions involved in xenobiotic biotransformation using the available information on metabolites, enzymes, and gut bacteria from various publicly available databases discussed in the above section (Gupta et al., 2014; Sharma et al., 2017b; Srivastava et al., 2020). Basic workflow for developing machine learning-based tools involves data collection, generation of features for the data and selecting most important features, training different machine learning algorithms to identify best performing classifier, and optimization of the selected algorithm to further improve its performance (Srivastava et al., 2020; Gupta et al., 2022; Tomer et al., 2023).

For successful development of AI/ML-based xenobiotic metabolism prediction tools, a curated dataset that includes substrate information and their labels indicating the EC class of the substrate is required. This dataset is generally split into 80:20 ratio where training set holds 80% of the data and the remaining 20% data is used as the blind set (Joseph, 2022). Training set is used for training and comparing the performance of different algorithms to select the best performing algorithm, for feature selection and for optimization of classifier model, whereas the blind set is used to test the performance of optimised models. Model training is performed using k-fold cross validation method, where the training dataset is split equally into k-folds/parts and training is carried out in k-number of iterations (Sechidis et al., 2011; Ali et al., 2012). For each iteration, one-fold is kept as testing set while the other folds are used for training, and the whole process is carried out such that each fold is used as testing set across the k-number of iterations.

Finally, to validate the performance of trained models, a validation dataset can be used, which has no overlap with the training dataset. This step ensures that the developed machine learning model shows no bias and can be generalized across different datasets/samples after its deployment. To develop ML models for predicting the gut bacteria mediated xenobiotic biotransformation, substrate information for enzymes can be used for training to predict complete EC number of all the gut bacterial enzymes. Prediction of complete EC number helps to identify the complete xenobiotic biotransformation reaction and the involved gut bacteria (Figure 1B).

## Generation of different types of features

Features are the set of variables that explain different properties of the data and are among the most important aspects for training and development of prediction models (Blum and Langley, 1997). In case of molecular/substrate data, variables that provide information regarding the chemical, physical or structural properties for each substrate can be used as features (Figure 1A). Using the chemical information for xenobiotic molecules, different types of features can be generated that can be used for developing chemoinformatic and machine learning-based prediction models (Sharma et al., 2017a; Malwe et al., 2023). Molecular descriptors are set of features generated based on the physical and chemical properties like molecular weight, charge, valency, etc., for molecules, which can be generated using RDKit (RDKit: Open-source cheminformatics; http://www.rdkit.org). RDKit is an open source library that is widely used in chemoinformatic analysis that can be used to perform different tasks such as feature generation, reading various molecule file types (such as .sdf, .mol, etc.), interconversion of molecule file types etc. Similarly, fingerprints are the set of features that provide structural information for the molecules. Fingerprints are set of binary features that describe the structure of a molecule including number of atoms, different types of bonds etc. Different types of fingerprints such as extended connectivity fingerprints, linear and circular fingerprints can be generated using PaDeL (Yap, 2011) or RDKit. Recently, neural networks-based approach such as directed message passing neural networks (DMPNN) was applied to obtain molecular features (Stokes et al., 2020). Combining the human gut microbiome information and chemical information for xenobiotics that are metabolised by gut bacteria, computational models can be developed to predict the potential xenobiotic biotransformation and provide lead candidates for experimental validations.

## Feature selection

Out of all the generated features, it is important to note that not all features are relevant for making accurate predictions. Therefore, it is important to select the most important features in molecules such that the performance of models is enhanced (or not negatively affected). This is known as feature selection that helps in selecting the most important features as well as in reducing the size of the models, which improves their speed and efficiency (Sharma et al., 2015; Jaiswal et al., 2021). There are multiple methods to perform feature selection such as Recursive Feature Elimination (RFE) (Chen and Jeong, 2007), k-best feature selection and Boruta based feature selection etc. RFE is wrapper style feature selection method that can be implemented using any supervised learning algorithm. RFE performs successive iterations in which it removes the least important features in each iteration till a pre-determined number of features remain. RFE is popular for its simplicity however it is computationally extensive and requires a specified number of features to be selected which is difficult to determine. On the other hand, Boruta based feature selection utilizes random forest-based classifier in which Boruta’s feature selection algorithm is used as a wrapper (Kursa and Rudnicki, 2010). Boruta generates shadow features for each feature present in the dataset and trains the RF classifier on it to calculate the feature importance measured using mean decrease in accuracy. Shadow feature for a particular feature is calculated by randomly shuffling values of the feature. Thus, one shadow feature for a real feature is generated. In the next step, feature importance for each shadow feature is calculated and same process is repeated using real features. After comparing feature importance of a real feature against its shadow feature, the feature is selected if its importance is higher than its shadow feature. Boruta selects any number of features it considers important and does not require any pre-determined number of features to be selected.

Since different types of features can be generated for the molecules in a dataset (e.g., molecular descriptors, linear and circular fingerprints for substrate/molecule data), feature selection can be applied to each type of features independently to select most important features from each set of features. Combining different types of selected features to create a “hybrid features” set helps in combining different aspects for each sample in the data thus enhancing and maximising the information to be used for training. Such approach has been used for developing different types of ML-based classifiers (Sharma et al., 2017b; Srivastava et al., 2020).

## Parameter optimization and case study of amphetamine

Final step in developing AI/ML-based prediction model is optimizing the parameters of the best performing algorithm during training. Machine learning algorithms can be customized by modifying their parameters. Such customization, termed as parameter optimization helps in further improving the performance of the AI/ML models. Number of parameters and their functions vary among different algorithms. For example, in case of random forest, parameters determining number of trees in a random forest or number of features to be used during construction a single tree are the most widely optimised parameters (Sharma et al., 2017a; Srivastava et al., 2020). Similarly, for SVM, parameters such as C, gamma or kernel function that determine distance between classes and hyperplane, number of points to be classified correctly, and shape of hyperplane are optimized to improve performance of SVM classifier (Gupta et al., 2022). Neural networks offer a large number of parameters that can be optimized to enhance classification accuracy of the prediction model. Using neural networks, number of hidden layers, number of nodes in individual hidden layers, the weightage for each node in the network can be optimised. Parameter optimization can be performed using GridSearchCV method (Ahmad et al., 2022) available on python as well as R, which takes a list of values for each parameter to be optimised as an input and can compute any evaluation metric of choice. Using the input values, it returns a grid of values based on the combination of different values for the parameters, from which the best combination of values for parameters can be selected. Using parameter optimization, performance of the models can be enhanced further and help overcome issues such as overfitting (Ying, 2019).

One example of a molecule is Amphetamine that highlight the application of machine learning in predicting the enzymes that can metabolize this drug molecule, followed by validation through molecular dynamics approaches. Amphetamine is an FDA-approved central nervous system stimulant that is known to be metabolised by human gut bacterial tyramine oxidase (Hacisalihoglu et al., 2000). To predict gut bacterial enzymes involved in metabolism of amphetamine, the first step is to retrieve its .sdf file containing its structural information from PubChem (Kim et al., 2023). In the next step, various features such as molecular descriptors specifying its molecular properties such as atomic weight, valency, electron distribution, number of charged/uncharged functional groups etc. can be generated using RDKit. Similarly, various structural features such as linear and circular fingerprints of amphetamine can also be generated using PaDeL and RDKit, respectively. Once all the required features are generated, this feature table for amphetamine can be provided as input into a trained AI/ML classifier that will predict the gut bacterial enzymes involved in amphetamine biotransformation or bioaccumulation. Some tools that can help a user to perform the above steps are DrugBug (Sharma et al., 2017a) and GutBug (Malwe et al., 2023) and drug depletion predictor developed by McCoubrey et al. (2021). The prediction results can be further confirmed using molecular dynamics simulations (Kumar et al., 2019) and/or experimental methods. For example, in the case of Amphetamine, the molecular dynamics simulation revealed that it binds to tyramine oxidase from the *Escherichia coli* strain present in the human gut microbiome at the binding site that harbour polar and nonpolar amino acids, and validated the predicted promiscuous metabolism of amphetamine by a gut enzyme. Such validations help to improve the efficacy of the drug during the drug design and development process.

## Tools to predict microbiome-mediated biotransformation and bioaccumulation of xenobiotics

Using the available biological and chemical data and the various computational and AI/ML approaches, multiple predictions tools have been developed to predict the xenobiotic molecules prone to gut microbial biotransformation, the enzymes involved in biotransformation, complete reaction, and the gut bacterial species that harbour these xenobiotic metabolising enzymes. In addition, paired metagenome and metabolome data can also be used to understand differential metabolite abundance profile for a given metagenomic data (Table 2).

**Table 2:** Principle, advantages and disadvantages of different types of tools covered in this review.

Tools	Prediction method principle	Type of data used for training	Advantages	Disadvantages
Drug metabolism prediction tool (Mallory et al., 2018)	Chemical reaction vector embedding method	Biochemical reactions and metabolite information	Predicts EC number and gene ontology for predicted biochemical reactions	Requires drug and its converted metabolite information, thus trained on limited data
Mangosteen pipeline	Reference database-based method	KEGG orthologs and BioCyc reactions linked to microbiome data	Predicts generation or depletion of metabolites for given microbiome profile	Lower performance on predicting differential metabolite profiles
MIMOSA	Reference database-based method	Gene abundance data linked to microbiome abundance profiles	Predicts generation or depletion of metabolites for given microbiome profile	Performance on predicting differential metabolite profiles observed to be highly variable
MIMOSA2	Reference database-based method	Taxon specific metabolic models linked to KEGG	Predicts generation or depletion of metabolites for given microbiome profile	Predictions observed to be hampered due to *in vivo* effects
DeepEC	Convolutional neural networks-based prediction	Protein sequences	Predicts EC number and function for any protein sequence	Not trained to predict xenobiotic metabolism
MelonPann	Machine learning-based prediction	Paired metabolome-metagenome data	Predicts metabolome profile for any metagenomic data	Not tested for predicting metabolism of specific xenobiotic molecules
DrugBug	Machine learning-based prediction	Substrate and enzyme information	Predicts EC numbers for gut bacterial enzymes involved in biotransformation of xenobiotic molecules	No information regarding products of the predicted biotransformation. Incomplete EC numbers are predicted in some cases
GutBug	Machine learning-based prediction	Substrate and enzyme information	Predicts complete EC numbers for gut bacterial enzymes involved in biotransformation of biotic and xenobiotic molecules	No information regarding products of the predicted biotransformation.
Drug depletion prediction tool (McCoubrey et al., 2021)	Machine learning-based prediction	Drug molecule information	Predicts drug depletion due to human gut bacteria mediated biotransformation and bioaccumulation	No information regarding gut bacteria or gut bacterial enzymes involved in drug depletion

Various reference database-based tools such as Mangosteen (Yin et al., 2020), MIMOSA (Noecker et al., 2016) and MIMOSA2 (Noecker et al., 2022) integrate microbiome and metabolome data to predict presence or absence of metabolites for any given microbiome taxonomic or functional profile. Mangosteen pipeline uses functional profiles for microbiome that is linked to KEGG orthologs and BioCyc reactions (Karp et al., 2019) to predict occurrence of metabolites and differential metabolite profiles. Using data from six studies providing accessible high-quality data, Mangosteen was able to predict occurrence of 3,315 KEGG associated metabolites and 5,957 BioCyc associated metabolites which were then linked to their biological roles and metabolic pathways. However, Mangosteen performed poorly in identifying differentially abundant metabolites (Yin et al., 2020).

Similarly, MIMOSA is another reference database-based framework that uses gene abundance data for a microbiome profile to predict differential metabolic profiles (Noecker et al., 2016). Gene abundance data is mapped to its metabolic reference database that links enzymes with substrates and products. Using metabolic network model, MIMOSA calculates community-based metabolic potential (CMP) scores that measures the relative capacity of a microbial community to deplete or synthesize individual metabolites. MIMOSA framework was tested on two studies characterizing vaginal microbiome of healthy women and women with bacterial vaginosis. MIMOSA performed well to identify differentially abundant metabolites in healthy and bacterial vaginosis samples, however, for other studies that contained linked metabolome-metagenome data, MIMOSA performed poorly to identify differential metabolite profiles (Yin et al., 2020).

MIMOSA2 (Noecker et al., 2022) is a further improvement of the MIMOSA framework. MIMOSA2 assembles taxon-specific community metabolic models that is constructed by integrating microbiome data with reference databases such as KEGG. Based on the abundance profiles for each taxon in the community and genes available in the community metabolic models, CMP scores for each metabolite is calculated. In the next step, MIMOSA2 utilizes regression models to fit calculated CMPs with metabolomics data to infer taxon associated with generation or depletion of metabolites. Predictions by MIMOSA2 depend on qualitative information provided by reference databases and thus *in vivo* effects such as regulation and selection can hamper its performance (Noecker et al., 2022).

MelonnPan (Mallick et al., 2019) is another machine learning-based tool that predicts metabolomic profile of any input metagenomic data for which metabolome data is not yet available. MelonnPan uses paired metagenome-metabolome data for training per-metabolite elastic net regularized regression model. This regression model narrows down to minimal set of microbial features whose abundance profiles can be used to predict the particular metabolite. MelonnPan was validated on IBD metagenomic dataset and could predict diverse groups of metabolites such as sphingolipids, vitamin B-complex and bile acid derivatives (Mallick et al., 2019). Even though MelonnPan has tremendous applications in understanding metabolite occurrence in disease models and to predict metabolomic profiles for metagenomic data, direct application of MelonnPan to understand gut microbe-mediated metabolism of drugs and xenobiotics is difficult.

Since the annotation or assignment of EC numbers to metabolic enzymes is an important task while training the xenobiotic metabolism models, one such machine learning based tool to predict EC numbers is DeepEC (Ryu et al., 2019) that uses convolutional neural networks (CNN) to predict EC numbers for input protein sequences. DeepEC also performs homology-based search for predicted EC numbers and detects mutated domains in the protein sequence. Since the CNN model used in DeepEC is trained using protein sequence, it shows high precision and accuracy to predict EC numbers for any given protein sequence, however it is not trained to identify substrate-protein interactions. As a result, to predict and understand biotransformation of xenobiotic molecules using machine learning and other computational methods, other specialised tools are required which are mentioned below.

Using chemical reaction vector embedding method, Mallory et al. (2018) developed a pipeline to predict drug metabolism by human gut microbiome. In this study, biochemical reactions are treated as chemical transformations that are converted to algebraic expressions based on the change in chemical structure from conversion of substrate to products. This information is then used for predicting drug metabolism. Using 11,893 metabolites from 5,241 reactions, chemical vector space for each metabolite was calculated using RDKit and Tanimoto similarity search. In the next step, this chemical vector space is applied for reaction-level analysis to obtain difference vectors between various reactants and products. Finally, the drug-metabolite pairs were searched against this difference vector to identify the most similar reactions that can potentially be involved in conversion of queried drug into its metabolite. EC number and gene ontology for the predicted reactions were later retrieved to get complete information regarding the biochemical process involved in conversion of a drug into its metabolite. Using this approach, reactions for digoxin reduction and levodopa deamination were validated. This method however was developed using a limited number of drug-metabolite pairs and require information regarding the drug and its converted metabolite, thus not being able to predict potentially novel xenobiotic biotransformation.

DrugBug was one of the earliest machine learning-based tool that could predict gut bacterial species specific biotransformation of xenobiotic molecules (Sharma et al., 2017a). DrugBug utilized 324,697 metabolic enzymes from 491 human gut bacteria. For these enzymes, 1,609 substrates were utilized for training the machine learning models to predict EC number of the enzymes that can potentially metabolise the xenobiotic molecules. DrugBug performs classification using reaction class and reaction subclass specific random forest models developed in R to predict the EC class and EC subclass of enzymes. The substrate dataset used for training was highly diverse, thus an option to use two different models trained using up-sampled data and without up-sampled data is provided. DrugBug reported average 10-fold cross validation accuracy of 0.98 and accuracy of 0.93 on blind set. Since it was among the early tools, it was trained on a considerably lower amount of data since the knowledge of xenobiotics undergoing biotransformation was limited.

Another tool named GutBug was later developed that uses an improved rationale of predicting EC numbers of gut bacterial metabolic enzymes for prediction of EC class and EC subclass, GutBug is trained on 3,457 substrates belonging to diverse reaction classes. GutBug includes 12 independent random forest and artificial neural networks-based binary classifiers and 6 random forest-based multilabel models that predict reaction class and subclass for the input molecule. Complete reaction is predicted using KNN-based molecular similarity search. The gut bacterial enzymes database used in GutBug consisted of 363,872 metabolic enzymes from 690 gut bacterial strains. GutBug displayed accuracies between 0.78 and 0.97 across different reaction class and subclasses. Another key feature of GutBug is the identification of reaction centres within the query molecule that are prone to metabolism by the predicted enzymes for the RDM patterns database available on KEGG was used. In addition, GutBug also provides a pipeline for directly identifying predicted enzymes in metagenome assembled genomes (MAGs) (Malwe et al., 2023). The performance was validated on 27 molecules including xenobiotics/drugs as well as biotic molecules like bioactive dietary components/nutraceuticals. Thus, deeper insights into gut bacteria mediated biotransformation of xenobiotics and drugs can be obtained using chemical reaction vector embedding pipeline, DrugBug and GutBug, to get leads on the key gut bacterial enzymes and the species harbouring them.

Besides DrugBug and GutBug, a new classifier developed by McCoubrey et al., uses a different approach to predict small molecule drugs prone to gut microbe-mediated biotransformation. In this study, the authors grouped biotransformation and bioaccumulation together under the term “depleted” and developed a binary classifier (McCoubrey et al., 2021). The classifier was trained on 455 drugs and optimised extra trees classifier that provides a binary output on whether a drug is predicted to be depleted or not along with a prediction score. This classifier provides information on gut microbe-mediated biotransformation as well as bioaccumulation, however no information regarding specific gut bacteria or enzymes involved in depletion of small molecule drugs is provided. Moreover, number of known depleted molecules used for the training were limited. Further understanding of bioaccumulation process will shed light on newer cases of xenobiotics that are bioaccumulated by gut bacteria. As a result, by utilising updated databases, more detailed and sophisticated models can be developed to predict gut bacteria mediated bioaccumulation.

## Discussion

The ability of human gut microbiome to metabolise and accumulate xenobiotics have substantial health implications on the human host. Drug inactivation and accumulation within bacterial cells leads to depletion and loss of bioavailability of drugs whereas increased toxicity due to gut microbe-mediated biotransformation of xenobiotics can lead to unintended harmful effects on the host. In contrast, gut bacterial metabolism of xenobiotics can also lead to conversion of prodrug into its active form that could have beneficial outcomes. Thus, human gut microbiome mediated biotransformation of xenobiotics poses a risk as well as opportunity for drug development and modulation of human health. Similarly, AI/ML methods can also help in development of novel probiotics and prebiotics by predicting prebiotics/nutraceutical metabolising gut bacteria.

Dynamic nature of the gut microbiome and complexity arising out of it makes experimental identification of xenobiotic biotransformation/bioaccumulation by gut microbes a difficult task. However, using the available novel computational approaches and prediction tools, obtaining leads on possible microbial biotransformation of any drug under development now appears feasible. Such preliminary predictions also provide valuable information on exploring the structural changes in a drug molecule to protect the biotransformation-prone side group from the unintended or promiscuous microbial metabolism. AI-based prediction tools can also be useful to identify previously unknown gut microbial biotransformation and specific gut bacteria involved in depletion or biotransformation of drugs. For example, GutBug was able to predict previously unreported hydrolysis of zonisamide, a known anticonvulsant, performed by gut bacterial genera like *Delftia, Lysinibacillus, Cronobacter* etc. Similarly, for known bifidogenic prebiotics such as Lactosucrose, bifidobacterial enzymes involved in lactosucrose metabolism were not known (Ose et al., 2018). GutBug predicted sialidase, beta-fructofuranosidase and other hydrolase class enzymes that can metabolise lactosucrose (Malwe et al., 2023).

**Figure 3:**
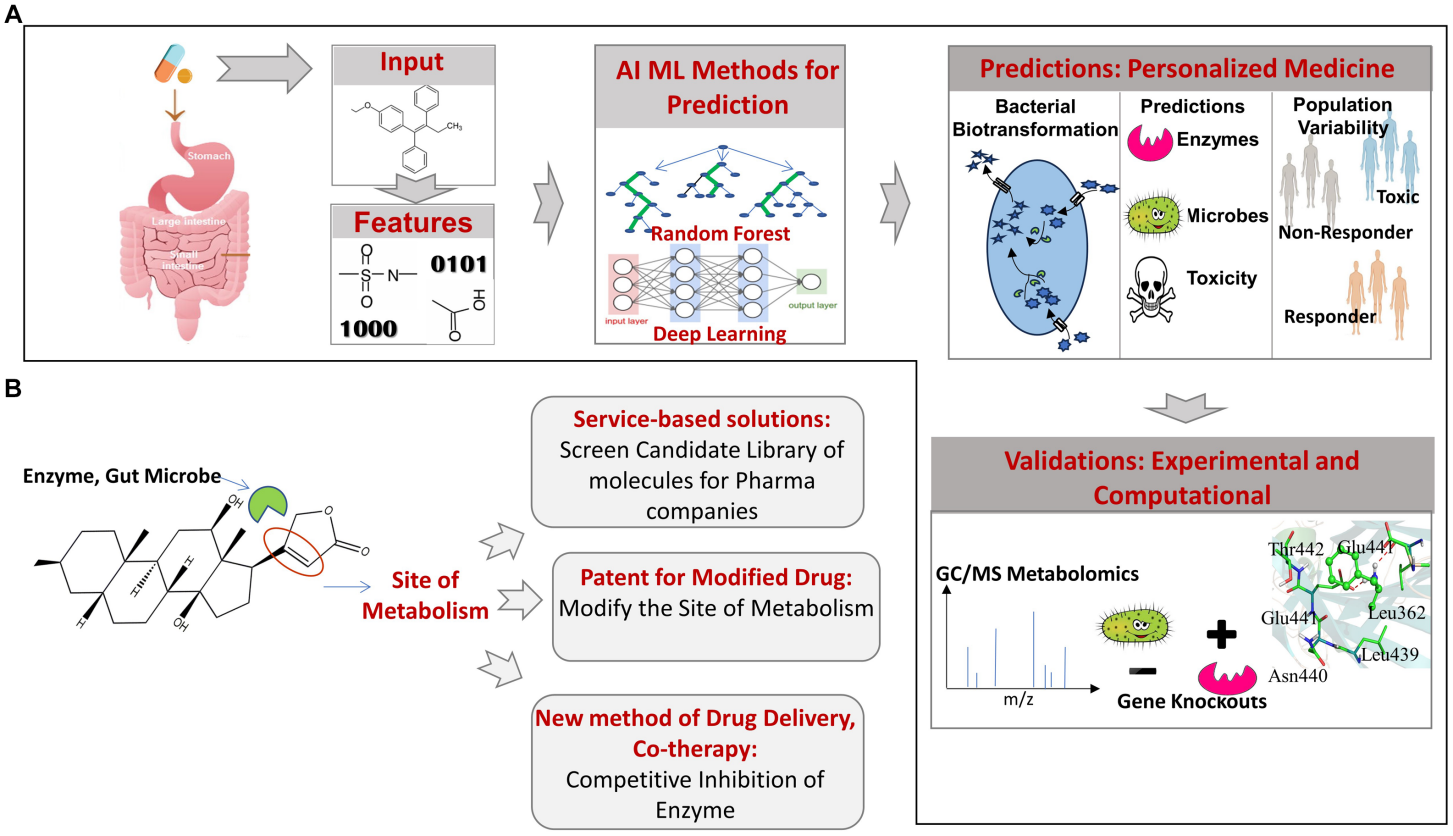
**(A)** Overview of AI/ML pipeline used for predicting gut bacteria-mediated xenobiotic biotransformation. **(B)** Applications of AI/ML prediction tools candidate drug screening, personalized medicines and drug delivery.

The above examples highlight the importance of the emerging computational approaches in obtaining novel insights into the gut microbiome mediated biotransformation, bioaccumulation, and depletion of orally administered xenobiotics. Employing prediction tools for rapid screening of library of candidate drugs molecules can help to identify molecules that are more susceptible to microbial degradation and depletion. Such insights can help in redesigning the structure of susceptible drug molecules to make them resistant to promiscuous microbial biotransformation or formulating efficient dosage after factoring for drug depletion due to the action of gut microbes. Since, the development of drug and bringing it to the market is an expensive and time taking process, a prior knowledge of the promiscuous metabolism is likely to save cost and time. Another key application of using prediction tools would be in personalised medication. In addition to the gut bacteria and the metabolic enzyme, the information on the populations or individuals that have an abundance of such bacteria will be very much needed and can be obtained by the metagenomic profiling of individuals, which can be used to prescribe the medication as per the individual or population-specific metagenomic profiles (Figure 3).

**Figure 4:**
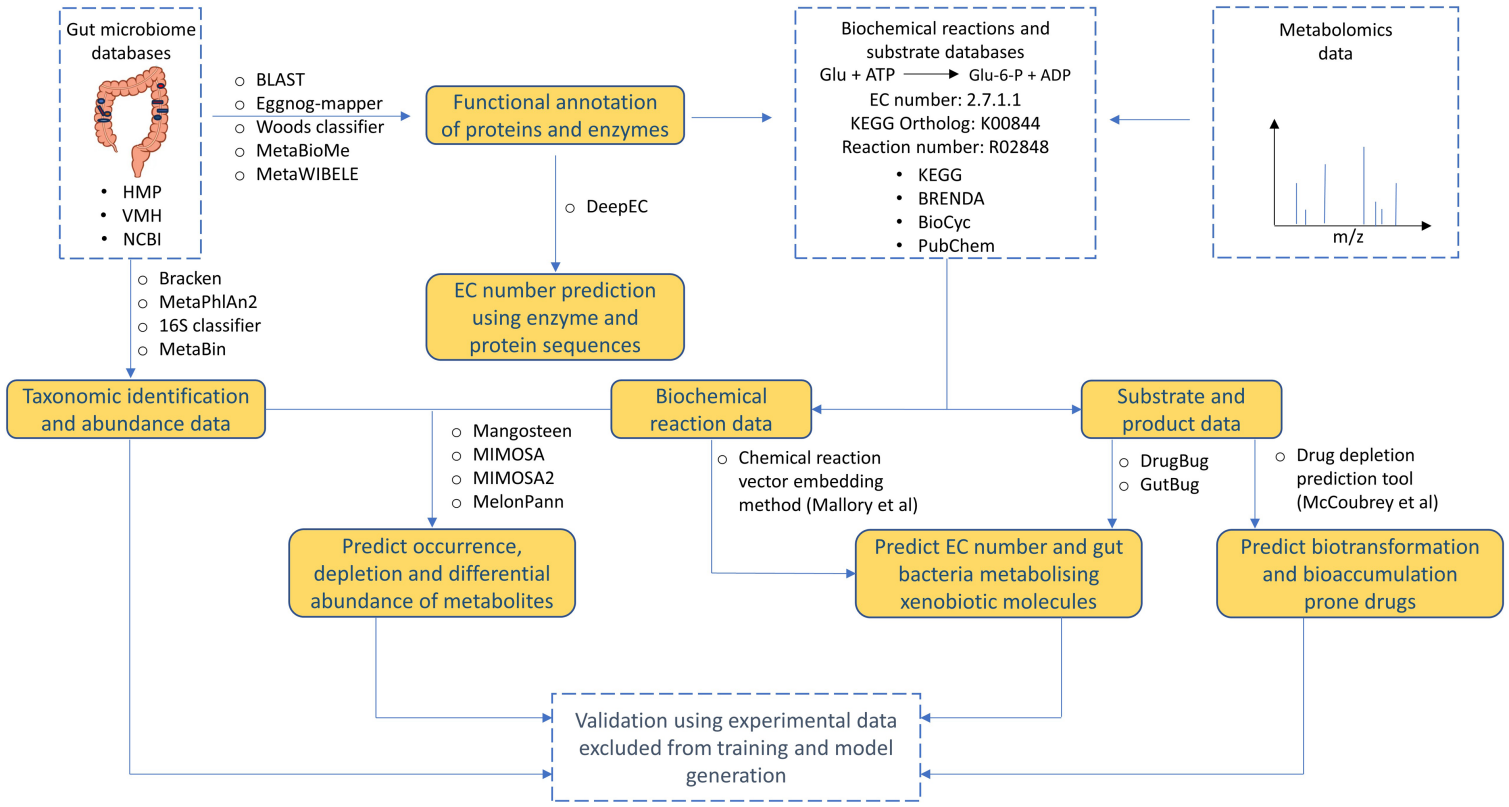
Overview of tools and databases useful for processing and deriving different types of information from human gut microbiome data.

Currently, various tools are available that can be used to decipher and understand different aspects of xenobiotic metabolism by human gut microbiome (Figure 4, Table 2). Reference database-based tools such as Mangosteen and MIMOSA that identify depletion or occurrence of metabolites based on metagenomic data can help potentially help in identifying which drug molecules are prone to metabolism. However, since reference databases are not updated to accommodate promiscuous metabolism by gut bacterial enzymes, there is high probability of missing out on identifying important xenobiotic metabolisms. Similarly, ML-based tools such as MelonnPan that uses paired metabolome-metagenome data can provide limited information for metabolism of specific xenobiotic molecules. Other AI/ML-based tools such as DrugBug and GutBug can predict promiscuous metabolism that have not been previously identified but can only provide qualitative predictions of gut bacterial enzymes involved in drug metabolism. Moreover, almost all the available tools suffer from unannotated or characterized genes. Available tools need to continuously update their training models based on updated annotations in reference databases that poses a challenging task. Recently, frameworks such as MetaWIBELE (Zhang et al., 2022) that predict function for uncharacterized genes in metagenomic datasets can be helpful in this regard. Similar frameworks can be integrated into current or new tools to account for unannotated proteins. Lastly, the use of extensive validation set for validating performance of tools predicting xenobiotic metabolism helps in eliminating bias and substantiating performance of models obtained during training. Validation set also helps in determining how models can perform on real data outside of training data and comparing performance against other tools.

International efforts to study human gut microbiome at holistic as well as at population level will provide further leads into the diverse mechanisms and interactions within the gut microbiome community, thus providing more information on the xenobiotics undergoing biotransformation as well as enzymes harboured by microbes (Turnbaugh et al., 2007; Integrative HMP (iHMP) Research Network Consortium, 2019). Further improvements in feature generation, machine learning and deep learning algorithms along with the discovery of new gut bacteria-xenobiotics interactions and reactions will provide unprecedented opportunities to develop robust prediction models. However, still the experimental validation of the predicted gut bacteria-metabolite interactions, and gut bacterial biotransformation of specific xenobiotics remains the gold standard and is needed to confirm the computational predictions. Therefore, it can be concluded that the AI-based computational approaches are promising in predicting the gut bacterial biotransformation of xenobiotics and provide leads for experimental validations while reducing the gigantic effort to experimentally determine all such biotransformation and validating only the predicted cases of metabolism. Thus, an integration of both computational and experimental approaches will provide deeper insights to understand how gut microbiome play an important role in xenobiotic metabolism in respect to the human health.

## Author contributions

AM: Writing – original draft, Writing – review & editing. VS: Writing – original draft, Writing – review & editing.

## References

[ref1] Abbruzzese J. L. Grunewald R. Weeks E. A. Gravel D. Adams T. Nowak B.. (1991). A phase I clinical, plasma, and cellular pharmacology study of gemcitabine. J. Clin. Oncol. 9, 491–498. doi: 10.1200/JCO.1991.9.3.491 1999720

[ref2] Ahmad G. N. Fatima H. Ullah S. Saidi A. S. (2022). Efficient medical diagnosis of human heart diseases using machine learning techniques with and without GridSearchCV. IEEE Access 10, 80151–80173. doi: 10.1109/ACCESS.2022.3165792

[ref3] Alam A. N. Saha J. R. Dobkin J. F. Lindenbaum J. (1988). Interethnic variation in the metabolic inactivation of digoxin by the gut flora. Gastroenterology 95, 117–123. doi: 10.1016/0016-5085(88)90299-5, PMID: 3371607

[ref4] Ali J. Khan R. Ahmad N. Maqsood I. (2012). Random forests and decision trees. Int. J. Comput. Sci. Issues 9:272. Available at: www.IJCSI.org

[ref5] Almeida A. Nayfach S. Boland M. Strozzi F. Beracochea M. Shi Z. J.. (2021). A unified catalog of 204,938 reference genomes from the human gut microbiome. Nat. Biotechnol. 39, 105–114. doi: 10.1038/s41587-020-0603-3, PMID: 32690973 PMC7801254

[ref6] Babtie A. Tokuriki N. Hollfelder F. (2010). What makes an enzyme promiscuous? Curr. Opin. Chem. Biol. 14, 200–207. doi: 10.1016/j.cbpa.2009.11.028 20080434

[ref7] Bajusz D. Rácz A. Héberger K. (2015). Why is Tanimoto index an appropriate choice for fingerprint-based similarity calculations? J. Chem. 7:20. doi: 10.1186/s13321-015-0069-3, PMID: 26052348 PMC4456712

[ref8] Batra M. Agrawal R. (2018). “Comparative analysis of decision tree algorithms” in Nature inspired computing. eds. Panigrahi B. Hoda M. Sharma V. Goel S. (Singapore: Springer), 31–36.

[ref9] Blandino G. Inturri R. Lazzara F. di Rosa M. Malaguarnera L. (2016). Impact of gut microbiota on diabetes mellitus. Diabetes Metab. 42, 303–315. doi: 10.1016/j.diabet.2016.04.004 27179626

[ref10] Blum A. L. Langley P. (1997). Selection of relevant features and examples in machine learning. Artif. Intell. 97, 245–271. doi: 10.1016/S0004-3702(97)00063-5

[ref11] Brandi G. Dabard J. Raibaud P. di Battista M. Bridonneau C. Pisi A. M.. (2006). Intestinal microflora and digestive toxicity of irinotecan in mice. Clin. Cancer Res. 12, 1299–1307. doi: 10.1158/1078-0432.CCR-05-0750, PMID: 16489087

[ref12] Brandt L. J. (2013). American journal of gastroenterology lecture: intestinal microbiota and the role of fecal microbiota transplant (FMT) in treatment of *C. difficile* infection. Am. J. Gastroenterol. 108, 177–185. doi: 10.1038/ajg.2012.450, PMID: 23318479

[ref13] Cantalapiedra C. P. Hernández-Plaza A. Letunic I. Bork P. Huerta-Cepas J. (2021). eggNOG-mapper v2: functional annotation, Orthology assignments, and domain prediction at the metagenomic scale. Mol. Biol. Evol. 38, 5825–5829. doi: 10.1093/molbev/msab293, PMID: 34597405 PMC8662613

[ref14] Carmody R. N. Turnbaugh P. J. (2014). Host-microbial interactions in the metabolism of therapeutic and diet-derived xenobiotics. J. Clin. Investig. 124, 4173–4181. doi: 10.1172/JCI72335, PMID: 25105361 PMC4191041

[ref15] Cencic A. Chingwaru W. (2010). The role of functional foods, nutraceuticals, and food supplements in intestinal health. Nutrients 2, 611–625. doi: 10.3390/nu2060611, PMID: 22254045 PMC3257668

[ref16] Chaudhary N. Sharma A. K. Agarwal P. Gupta A. Sharma V. K. (2015). 16S classifier: a tool for fast and accurate taxonomic classification of 16S rRNA hypervariable regions in metagenomic datasets. PLoS One 10:e0116106. doi: 10.1371/journal.pone.0116106, PMID: 25646627 PMC4315456

[ref17] Chen X. Jeong J. C. (2007) ‘Enhanced recursive feature elimination’, in *Sixth International Conference on Machine Learning and Applications (ICMLA 2007)*. IEEE, pp. 429–435.

[ref18] Chen R. Li L. Weng Z. (2003). ZDOCK: an initial-stage protein-docking algorithm. Proteins 52, 80–87. doi: 10.1002/prot.10389, PMID: 12784371

[ref19] Cheng F. Li W. Zhou Y. Shen J. Wu Z. Liu G.. (2012). admetSAR: a comprehensive source and free tool for assessment of chemical ADMET properties. J. Chem. Inf. Model. 52, 3099–3105. doi: 10.1021/ci300367a, PMID: 23092397

[ref20] Cherkasov A. Muratov E. N. Fourches D. Varnek A. Baskin I. I. Cronin M.. (2014). QSAR modeling: where have you been? Where are you going to? J. Med. Chem. 57, 4977–5010. doi: 10.1021/jm4004285, PMID: 24351051 PMC4074254

[ref21] Clare A. King R. D. (2001). “Knowledge discovery in multi-label phenotype data” in Principles of data mining and knowledge discovery. eds. De Raedt L. Siebes A. (Berlin: Springer), 42–53.

[ref22] Cohen Z. Kelly L. (2022). Bioaccumulation as a mechanism of microbiome/drug interactions. Trends Microbiol. 30, 99–101. doi: 10.1016/j.tim.2021.12.003, PMID: 34952771

[ref23] Collins S. L. Patterson A. D. (2020). The gut microbiome: an orchestrator of xenobiotic metabolism. Acta Pharm. Sin. B 10, 19–32. doi: 10.1016/j.apsb.2019.12.001, PMID: 31998605 PMC6984741

[ref24] Cornish-Bowden A. (2014). Current IUBMB recommendations on enzyme nomenclature and kinetics. Perspect. Sci. 1, 74–87. doi: 10.1016/j.pisc.2014.02.006

[ref25] Crouwel F. Buiter H. J. C. de Boer N. K. (2021). Gut microbiota-driven drug metabolism in inflammatory bowel disease. J. Crohn's Colitis 15, 307–315. doi: 10.1093/ecco-jcc/jjaa143, PMID: 32652007 PMC7904070

[ref26] Culp E. J. Goodman A. L. (2023). Cross-feeding in the gut microbiome: ecology and mechanisms. Cell Host Microbe 31, 485–499. doi: 10.1016/j.chom.2023.03.016, PMID: 37054671 PMC10125260

[ref27] Cutler A. Cutler D. R. Stevens J. R. (2012). “Random forests” in Ensemble machine learning. eds. Zhang C. Ma Y. Q. (Springer New York: New York, NY), 157–175.

[ref28] Dalianis H. (2018). “Evaluation metrics and evaluation” in Clinical text mining. ed. Dalianis H. (Cham: Springer), 45–53.

[ref29] Elisseeff A. Weston J. (2002). “A kernel method for multi-labelled classification” in *Advances in neural information processing systems* [Preprint]. eds. Dietterich T. G. Becker S. Ghahramani Z. (Cambridge, MA: The MIT Press).

[ref30] Enright E. F. Gahan C. G. Joyce S. A. Griffin B. T. (2016). The impact of the gut microbiota on drug metabolism and clinical outcome. Yale J. Biol. Med. 89, 375–382. PMID: 27698621 PMC5045146

[ref31] Eschenmoser A. (1995). One hundred years lock-and-key principle. Angew. Chem. Int. Ed. Engl. 33:2363. doi: 10.1002/anie.199423631

[ref32] Fan J. Fu A. Zhang L. (2019). Progress in molecular docking. Quant. Biol. 7, 83–89. doi: 10.1007/s40484-019-0172-y

[ref33] Frese K. K. Neesse A. Cook N. Bapiro T. E. Lolkema M. P. Jodrell D. I.. (2012). Nab-paclitaxel potentiates gemcitabine activity by reducing cytidine deaminase levels in a mouse model of pancreatic cancer. Cancer Discov. 2, 260–269. doi: 10.1158/2159-8290.CD-11-0242, PMID: 22585996 PMC4866937

[ref34] Geller L. T. Barzily-Rokni M. Danino T. Jonas O. H. Shental N. Nejman D.. (2017). Potential role of intratumor bacteria in mediating tumor resistance to the chemotherapeutic drug gemcitabine. Science 357, 1156–1160. doi: 10.1126/science.aah5043, PMID: 28912244 PMC5727343

[ref35] Gevers D. Pop M. Schloss P. D. Huttenhower C. (2012). Bioinformatics for the human microbiome project. PLoS Comput. Biol. 8:e1002779. doi: 10.1371/journal.pcbi.1002779, PMID: 23209389 PMC3510052

[ref36] Gomaa E. Z. (2020). Human gut microbiota/microbiome in health and diseases: a review. Antonie Van Leeuwenhoek 113, 2019–2040. doi: 10.1007/s10482-020-01474-7, PMID: 33136284

[ref37] Greener J. G. Kandathil S. M. Moffat L. Jones D. T. (2022). A guide to machine learning for biologists. Nat. Rev. Mol. Cell Biol. 23, 40–55. doi: 10.1038/s41580-021-00407-0, PMID: 34518686

[ref38] Guinane C. M. Cotter P. D. (2013). Role of the gut microbiota in health and chronic gastrointestinal disease: understanding a hidden metabolic organ. Ther. Adv. Gastroenterol. 6, 295–308. doi: 10.1177/1756283X13482996, PMID: 23814609 PMC3667473

[ref39] Guo Y. Lee H. Jeong H. (2020). Gut microbiota in reductive drug metabolism. Prog. Mol. Biol. Transl. Sci. 171, 61–93. doi: 10.1016/bs.pmbts.2020.04.002 32475528

[ref40] Gupta A. Kapil R. Dhakan D. B. Sharma V. K. (2014). MP3: a software tool for the prediction of pathogenic proteins in genomic and metagenomic data. PLoS One 9:e93907. doi: 10.1371/journal.pone.0093907, PMID: 24736651 PMC3988012

[ref41] Gupta S. Madhu M. K. Sharma A. K. Sharma V. K. (2016). ProInflam: a webserver for the prediction of proinflammatory antigenicity of peptides and proteins. J. Transl. Med. 14:178. doi: 10.1186/s12967-016-0928-3, PMID: 27301453 PMC4908730

[ref42] Gupta A. Malwe A. S. Srivastava G. N. Thoudam P. Hibare K. Sharma V. K. (2022). MP4: a machine learning based classification tool for prediction and functional annotation of pathogenic proteins from metagenomic and genomic datasets. BMC Bioinform. 23:507. doi: 10.1186/s12859-022-05061-7, PMID: 36443666 PMC9703692

[ref43] Guthrie L. Gupta S. Daily J. Kelly L. (2017). Human microbiome signatures of differential colorectal cancer drug metabolism. NPJ Biofilms Microbiomes 3:27. doi: 10.1038/s41522-017-0034-1, PMID: 29104759 PMC5665930

[ref44] Hacisalihoglu A. Jongejan A. Jongejan J. A. Duine J. A. (2000). Enantioselective oxidation of amphetamine by copper-containing quinoprotein amine oxidases from Escherichia coli and *Klebsiella oxytoca*. J. Mol. Catal. B Enzym. 11, 81–88. doi: 10.1016/S1381-1177(00)00216-2

[ref45] Haiser H. J. Gootenberg D. B. Chatman K. Sirasani G. Balskus E. P. Turnbaugh P. J. (2013). Predicting and manipulating cardiac drug inactivation by the human gut bacterium *Eggerthella lenta*. Science 341, 295–298. doi: 10.1126/science.1235872, PMID: 23869020 PMC3736355

[ref46] Haiser H. J. Seim K. L. Balskus E. P. Turnbaugh P. J. (2014). Mechanistic insight into digoxin inactivation by *Eggerthella lenta* augments our understanding of its pharmacokinetics. Gut Microbes 5, 233–238. doi: 10.4161/gmic.27915, PMID: 24637603 PMC4063850

[ref47] Hollingsworth S. A. Dror R. O. (2018). Molecular dynamics simulation for all. Neuron 99, 1129–1143. doi: 10.1016/j.neuron.2018.08.011, PMID: 30236283 PMC6209097

[ref48] Hult K. Berglund P. (2007). Enzyme promiscuity: mechanism and applications. Trends Biotechnol. 25, 231–238. doi: 10.1016/j.tibtech.2007.03.002, PMID: 17379338

[ref49] Hutchinson T. C. Hellebust J. A. Mackay D. Tarn D. Kauss P. (1979). Relationship of hydrocarbon solubility to toxicity in algae and cellular membrane effects. Int. Oil Spill Conf. Proc. 1979, 541–547. doi: 10.7901/2169-3358-1979-1-541

[ref50] Ilett K. F. Tee L. B. G. Reeves P. T. Minchin R. F. (1990). Mebolism of drugs and other xenobiotics in the gut lumen and wall. Pharmacol. Ther. 46, 67–93. doi: 10.1016/0163-7258(90)90036-2, PMID: 2181492

[ref51] Integrative HMP (iHMP) Research Network Consortium (2019). The integrative human microbiome project. Nature 569, 641–648. doi: 10.1038/s41586-019-1238-8, PMID: 31142853 PMC6784865

[ref52] Jaiswal S. K. Agarwal S. M. Thodum P. Sharma V. K. (2021). SkinBug: an artificial intelligence approach to predict human skin microbiome-mediated metabolism of biotics and xenobiotics. iScience 24:101925. doi: 10.1016/j.isci.2020.101925, PMID: 33385118 PMC7772573

[ref53] Javdan B. Lopez J. G. Chankhamjon P. Lee Y. C. J. Hull R. Wu Q.. (2020). Personalized mapping of drug metabolism by the human gut microbiome. Cells 181, 1661–1679.e22. doi: 10.1016/j.cell.2020.05.001, PMID: 32526207 PMC8591631

[ref54] Jethwani P. Grover K. (2019). Gut microbiota in health and diseases – a review. Int. J. Curr. Microbiol. App. Sci. 8, 1586–1599. doi: 10.20546/ijcmas.2019.808.187

[ref55] Joseph V. R. (2022). Optimal ratio for data splitting. *Stat*. *Anal. Data Min* 15, 531–538. doi: 10.1002/sam.11583

[ref56] Jourova L. Anzenbacher P. Anzenbacherova E. (2016). Human gut microbiota plays a role in the metabolism of drugs. Biomed. Papers 160, 317–326. doi: 10.5507/bp.2016.039, PMID: 27485182

[ref57] Kanehisa M. (2000). KEGG: Kyoto encyclopedia of genes and genomes. Nucleic Acids Res. 28, 27–30. doi: 10.1093/nar/28.1.27, PMID: 10592173 PMC102409

[ref59] Karp P. D. Billington R. Caspi R. Fulcher C. A. Latendresse M. Kothari A.. (2019). The BioCyc collection of microbial genomes and metabolic pathways. Brief. Bioinform. 20, 1085–1093. doi: 10.1093/bib/bbx085, PMID: 29447345 PMC6781571

[ref60] Khersonsky O. Roodveldt C. Tawfik D. (2006). Enzyme promiscuity: evolutionary and mechanistic aspects. Curr. Opin. Chem. Biol. 10, 498–508. doi: 10.1016/j.cbpa.2006.08.011, PMID: 16939713

[ref61] Kim S. Chen J. Cheng T. Gindulyte A. He J. He S.. (2023). PubChem 2023 update. Nucleic Acids Res. 51, D1373–D1380. doi: 10.1093/nar/gkac956, PMID: 36305812 PMC9825602

[ref62] Klinger S. Austin J. (2006) ‘Weighted superstructures for chemical similarity searching’, *Proceedings of the 9th Joint Conference on Information Sciences*, pp. 1–19. Available at: http://www.cs.york.ac.uk/arch/publications/byyear/2006/2006_WeightedSuperstructuresForChemicalSimilarity_234.pdf.

[ref63] Klotz U. Maier K. Fischer C. Heinkel K. (1980). Therapeutic efficacy of sulfasalazine and its metabolites in patients with ulcerative colitis and Crohn’s disease. N. Engl. J. Med. 303, 1499–1502. doi: 10.1056/NEJM198012253032602, PMID: 6107853

[ref64] Klünemann M. Andrejev S. Blasche S. Mateus A. Phapale P. Devendran S.. (2021). Bioaccumulation of therapeutic drugs by human gut bacteria. Nature 597, 533–538. doi: 10.1038/s41586-021-03891-8, PMID: 34497420 PMC7614428

[ref65] Koppel N. Maini Rekdal V. Balskus E. P. (2017). Chemical transformation of xenobiotics by the human gut microbiota. Science 356:eaag2770. doi: 10.1126/science.aag2770, PMID: 28642381 PMC5534341

[ref66] Koshland D. E. (2004). Crazy, but correct. Nature 432:447. doi: 10.1038/432447a, PMID: 15565132

[ref67] Krogh A. (2008). What are artificial neural networks? Nat. Biotechnol. 26, 195–197. doi: 10.1038/nbt1386 18259176

[ref68] Kumar K. Dhoke G. V. Sharma A. K. Jaiswal S. K. Sharma V. K. (2019). Mechanistic elucidation of amphetamine metabolism by tyramine oxidase from human gut microbiota using molecular dynamics simulations. J. Cell. Biochem. 120, 11206–11215. doi: 10.1002/jcb.28396, PMID: 30701587

[ref69] Kumar K. Jaiswal S. K. Dhoke G. V. Srivastava G. N. Sharma A. K. Sharma V. K. (2018). Mechanistic and structural insight into promiscuity based metabolism of cardiac drug digoxin by gut microbial enzyme. J. Cell. Biochem. 119, 5287–5296. doi: 10.1002/jcb.26638, PMID: 29274283

[ref70] Kursa M. B. Rudnicki W. R. (2010). Feature selection with the Boruta package. J. Stat. Softw. 36, 1–3. doi: 10.18637/jss.v036.i11

[ref71] Lang P. T. Brozell S. R. Mukherjee S. Pettersen E. F. Meng E. C. Thomas V.. (2009). DOCK 6: combining techniques to model RNA–small molecule complexes. RNA 15, 1219–1230. doi: 10.1261/rna.1563609, PMID: 19369428 PMC2685511

[ref72] Li Y. Yu H. Chen Y. Lau K. Cai L. Cao H.. (2011). Substrate promiscuity of N-Acetylhexosamine 1-kinases. Molecules 16, 6396–6407. doi: 10.3390/molecules16086396, PMID: 21799473 PMC6264712

[ref73] Lu J. Breitwieser F. P. Thielen P. Salzberg S. L. (2017). Bracken: estimating species abundance in metagenomics data. PeerJ Comput. Sci. 3:e104. doi: 10.7717/peerj-cs.104 PMC12016282 40271438

[ref74] Mallick H. Franzosa E. A. Mclver L. J. Banerjee S. Sirota-Madi A. Kostic A. D.. (2019). Predictive metabolomic profiling of microbial communities using amplicon or metagenomic sequences. Nat. Commun. 10:3136. doi: 10.1038/s41467-019-10927-1, PMID: 31316056 PMC6637180

[ref75] Mallory E. K. Acharya A. Rensi S. E. Turnbaugh P. J. Bright R. A. Altman R. B. (2018). Chemical reaction vector embeddings: towards predicting drug metabolism in the human gut microbiome. Pac. Symp. Biocomput. 23, 56–67. doi: 10.1142/9789813235533_0006 29218869 PMC5771676

[ref76] Malwe A. S. Srivastava G. N. Sharma V. K. (2023). GutBug: a tool for prediction of human gut Bacteria mediated biotransformation of biotic and xenobiotic molecules using machine learning. J. Mol. Biol. 435:168056. doi: 10.1016/j.jmb.2023.168056, PMID: 37356904

[ref77] Mathan V. I. Wiederman J. Dobkin J. F. Lindenbaum J. (1989). Geographic differences in digoxin inactivation, a metabolic activity of the human anaerobic gut flora. Gut 30, 971–977. doi: 10.1136/gut.30.7.971, PMID: 2759492 PMC1434295

[ref78] McCoubrey L. E. Thomaidou S. Elbadawi M. Gaisford S. Orlu M. Basit A. W. (2021). Machine learning predicts drug metabolism and bioaccumulation by intestinal microbiota. Pharmaceutics 13:2001. doi: 10.3390/pharmaceutics13122001, PMID: 34959282 PMC8707855

[ref79] Misselwitz B. Strittmatter G. Periaswamy B. Schlumberger M. C. Rout S. Horvath P.. (2010). Enhanced CellClassifier: a multi-class classification tool for microscopy images. BMC Bioinform. 11:30. doi: 10.1186/1471-2105-11-30, PMID: 20074370 PMC2821321

[ref80] Morris G. M. Lim-Wilby M. (2008). Molecular docking. Methods Mol. Biol. 443, 365–382. doi: 10.1007/978-1-59745-177-2_19 18446297

[ref81] Nasteski V. (2017). An overview of the supervised machine learning methods. Horizons. B 4, 51–62. doi: 10.20544/HORIZONS.B.04.1.17.P05

[ref82] Noble W. S. (2006). What is a support vector machine? Nat. Biotechnol. 24, 1565–1567. doi: 10.1038/nbt1206-1565 17160063

[ref83] Noecker C. Eng A. Muller E. Borenstein E. (2022). MIMOSA2: a metabolic network-based tool for inferring mechanism-supported relationships in microbiome-metabolome data. Bioinformatics 38, 1615–1623. doi: 10.1093/bioinformatics/btac003, PMID: 34999748 PMC8896604

[ref84] Noecker C. Eng A. Srinivasan S. Theriot C. M. Young V. B. Jansson J. K.. (2016). Metabolic model-based integration of microbiome taxonomic and Metabolomic profiles elucidates mechanistic links between ecological and metabolic variation. mSystems 1:e00013-15. doi: 10.1128/mSystems.00013-15, PMID: 27239563 PMC4883586

[ref85] Noronha A. Modamio J. Jarosz Y. Guerard E. Sompairac N. Preciat G.. (2019). The virtual metabolic human database: integrating human and gut microbiome metabolism with nutrition and disease. Nucleic Acids Res. 47, D614–D624. doi: 10.1093/nar/gky992, PMID: 30371894 PMC6323901

[ref86] Ose R. Hirano K. Maeno S. Nakagawa J. Salminen S. Tochio T.. (2018). The ability of human intestinal anaerobes to metabolize different oligosaccharides: novel means for microbiota modulation? Anaerobe 51, 110–119. doi: 10.1016/j.anaerobe.2018.04.018, PMID: 29734011

[ref87] Pruitt K. D. (2004). NCBI reference sequence (RefSeq): a curated non-redundant sequence database of genomes, transcripts and proteins. Nucleic Acids Res. 33, D501–D504. doi: 10.1093/nar/gki025, PMID: 15608248 PMC539979

[ref88] Pushpa M. Karpagavalli S. (2017). Multi-label classification: problem transformation methods in Tamil phoneme classification. Procedia Comput. Sci. 115, 572–579. doi: 10.1016/j.procs.2017.09.116

[ref89] Roberfroid M. B. Van Loo J. A. E. Gibson G. R. (1998). The bifidogenic nature of chicory inulin and its hydrolysis products. J. Nutr. 128, 11–19. doi: 10.1093/jn/128.1.11, PMID: 9430596

[ref90] Ryu J. Y. Kim H. U. Lee S. Y. (2019). Deep learning enables high-quality and high-throughput prediction of enzyme commission numbers. Proc. Natl. Acad. Sci. 116, 13996–14001. doi: 10.1073/pnas.1821905116, PMID: 31221760 PMC6628820

[ref91] Sokol H. Landman C. Seksik P. Berard L. Montil M. Nion-Larmurier I.. (2020). Fecal microbiota transplantation to maintain remission in Crohn’s disease: a pilot randomized controlled study. Microbiome 8:12. doi: 10.1186/s40168-020-0792-5, PMID: 32014035 PMC6998149

[ref92] Scheer M. Grote A. Chang A. Schomburg I. Munaretto C. Rother M.. (2011). BRENDA, the enzyme information system in 2011. Nucleic Acids Res. 39, D670–D676. doi: 10.1093/nar/gkq1089, PMID: 21062828 PMC3013686

[ref93] Sechidis K. Tsoumakas G. Vlahavas I. (2011) *On the stratification of multi-label data*. Available at: http://bailando.sims.berkeley.edu/enron_email.html.

[ref94] Sharma A. K. Gupta A. Kumar S. Dhakan D. B. Sharma V. K. (2015). Woods: a fast and accurate functional annotator and classifier of genomic and metagenomic sequences. Genomics 106, 1–6. doi: 10.1016/j.ygeno.2015.04.001, PMID: 25863333

[ref95] Sharma A. K. Jaiswal S. K. Chaudhary N. Sharma V. K. (2017a). A novel approach for the prediction of species-specific biotransformation of xenobiotic/drug molecules by the human gut microbiota. Sci. Rep. 7:9751. doi: 10.1038/s41598-017-10203-6, PMID: 28852076 PMC5575299

[ref96] Sharma V. K. Kumar N. Prakash T. Taylor T. D. (2010). MetaBioME: a database to explore commercially useful enzymes in metagenomic datasets. Nucleic Acids Res. 38, D468–D472. doi: 10.1093/nar/gkp1001, PMID: 19906710 PMC2808964

[ref97] Sharma V. K. Kumar N. Prakash T. Taylor T. D. (2012). Fast and accurate taxonomic assignments of metagenomic sequences using MetaBin. PLoS One 7:e34030. doi: 10.1371/journal.pone.0034030, PMID: 22496776 PMC3319535

[ref98] Sharma A. K. Srivastava G. N. Roy A. Sharma V. K. (2017b). ToxiM: a toxicity prediction tool for small molecules developed using machine learning and chemoinformatics approaches. Front. Pharmacol. 8:880. doi: 10.3389/fphar.2017.00880, PMID: 29249969 PMC5714866

[ref99] Srivastava G. N. Malwe A. S. Sharma A. K. Shastri V. Hibare K. Sharma V. K. (2020). Molib: a machine learning based classification tool for the prediction of biofilm inhibitory molecules. Genomics 112, 2823–2832. doi: 10.1016/j.ygeno.2020.03.020, PMID: 32229287

[ref100] Stokes J. M. Yang K. Swanson K. Jin W. Cubillos-Ruiz A. Donghia N. M.. (2020). A deep learning approach to antibiotic discovery. Cells 180, 688–702.e13. doi: 10.1016/j.cell.2020.01.021, PMID: 32084340 PMC8349178

[ref101] Suez J. Zmora N. Zilberman-Schapira G. Mor U. Dori-Bachash M. Bashiardes S.. (2018). Post-antibiotic gut mucosal microbiome reconstitution is impaired by probiotics and improved by autologous FMT. Cells 174, 1406–1423.e16. doi: 10.1016/j.cell.2018.08.047, PMID: 30193113

[ref102] Suthaharan S. (2016) ‘Support vector machine’, in: Suthaharan S., ed., Machine learning models and algorithms for big data classification, pp. 207–235. Springer, Boston, MA

[ref103] Tarca A. L. Carey V. J. Chen X. W. Romero R. Drăghici S. (2007). Machine learning and its applications to biology. PLoS Comput. Biol. 3:e116. doi: 10.1371/journal.pcbi.0030116, PMID: 17604446 PMC1904382

[ref104] Taunk K. De S. Verma S. Swetapadma A. (2019) ‘A brief review of nearest neighbor algorithm for learning and classification’, in *2019 International Conference on Intelligent Computing and Control Systems (ICCS)*. IEEE, pp. 1255–1260.

[ref105] Thompson J. (2002). Purification and some properties of phospho-Î^2^-galactosidase from the gram-negative oral bacterium *Leptotrichia buccalis* ATCC 14201. FEMS Microbiol. Lett. 214, 183–188. doi: 10.1111/j.1574-6968.2002.tb11344.x, PMID: 12351228

[ref106] Tomer R. Patiyal S. Dhall A. Raghava G. P. S. (2023). Prediction of celiac disease associated epitopes and motifs in a protein. Front. Immunol. 14:1056101. doi: 10.3389/fimmu.2023.1056101, PMID: 36742312 PMC9893285

[ref107] Truong D. T. Franzosa E. A. Tickle T. L. Scholz M. Weingart G. Pasolli E.. (2015). MetaPhlAn2 for enhanced metagenomic taxonomic profiling. Nat. Methods 12, 902–903. doi: 10.1038/nmeth.3589, PMID: 26418763

[ref108] Tsoumakas G. Katakis I. Vlahavas I. (2006) ‘A review of multi-label classification methods’, *Proceedings of the 2nd ADBIS Workshop on Data Mining and Knowledge Discovery (ADMKD 2006)*, pp. 99–109.

[ref109] Turnbaugh P. J. Ley R. E. Hamady M. Fraser-Liggett C. M. Knight R. Gordon J. I. (2007). The human microbiome project. Nature 449, 804–810. doi: 10.1038/nature06244, PMID: 17943116 PMC3709439

[ref110] UniProt Consortium (2015). UniProt: a hub for protein information. Nucleic Acids Res. 43, D204–D212. doi: 10.1093/nar/gku989, PMID: 25348405 PMC4384041

[ref111] Usama M. Qadir J. Raza A. Arif H. Yau K. L. A. Elkhatib Y.. (2019). Unsupervised machine learning for networking: techniques, applications and research challenges. IEEE Access 7, 65579–65615. doi: 10.1109/ACCESS.2019.2916648

[ref112] van Breemen R. B. Li Y. (2005). Caco-2 cell permeability assays to measure drug absorption. Expert Opin. Drug Metab. Toxicol. 1, 175–185. doi: 10.1517/17425255.1.2.175 16922635

[ref113] Vande Voorde J. Vervaeke P. Liekens S. Balzarini J. (2015). *Mycoplasma hyorhinis* -encoded cytidine deaminase efficiently inactivates cytosine-based anticancer drugs. FEBS Open Bio 5, 634–639. doi: 10.1016/j.fob.2015.07.007, PMID: 26322268 PMC4541722

[ref114] Verdonk M. L. Cole J. C. Hartshorn M. J. Murray C. W. Taylor R. D. (2003). Improved protein-ligand docking using GOLD. Proteins 52, 609–623. doi: 10.1002/prot.10465 12910460

[ref115] Wilson I. D. Nicholson J. K. (2017). Gut microbiome interactions with drug metabolism, efficacy, and toxicity. Transl. Res. 179, 204–222. doi: 10.1016/j.trsl.2016.08.002, PMID: 27591027 PMC5718288

[ref116] Wu G. Zhu J. (2020). Multi-label classification: do hamming loss and subset accuracy really conflict with each other? Adv. Neural Inf. Proces. Syst. 33, 3130–3140.

[ref117] Yan X. Yu P. S. Han J. (2005) ‘Substructure similarity search in graph databases’, in *Proceedings of the 2005 ACM SIGMOD international conference on management of data*, New York, NY, USA: ACM, pp. 766–777.

[ref118] Yang F.-J. (2018) ‘An implementation of naive Bayes classifier’, in *2018 International Conference on Computational Science and Computational Intelligence (CSCI)*. IEEE, pp. 301–306.

[ref119] Yap C. W. (2011). PaDEL-descriptor: an open source software to calculate molecular descriptors and fingerprints. J. Comput. Chem. 32, 1466–1474. doi: 10.1002/jcc.21707, PMID: 21425294

[ref120] Yin X. Altman T. Rutherford E. West K. A. Wu Y. Choi J.. (2020). A comparative evaluation of tools to predict metabolite profiles from microbiome sequencing data. Front. Microbiol. 11:595910. doi: 10.3389/fmicb.2020.595910, PMID: 33343536 PMC7746778

[ref121] Ying X. (2019). An overview of overfitting and its solutions. J. Phys. Conf. Ser. 1168:022022. doi: 10.1088/1742-6596/1168/2/022022

[ref123] Yoo D.-H. Kim I. S. van le T. K. Jung I. H. Yoo H. H. Kim D. H. (2014). Gut microbiota-mediated drug interactions between lovastatin and antibiotics. Drug Metab. Dispos. 42, 1508–1513. doi: 10.1124/dmd.114.058354, PMID: 24947972

[ref124] Zhang Y. Bhosle A. Bae S. McIver L. J. Pishchany G. Accorsi E. K.. (2022). Discovery of bioactive microbial gene products in inflammatory bowel disease. Nature 606, 754–760. doi: 10.1038/s41586-022-04648-7, PMID: 35614211 PMC9913614

[ref125] Zhang M.-L. Zhou Z.-H. (2007). ML-KNN: a lazy learning approach to multi-label learning. Pattern Recogn. 40, 2038–2048. doi: 10.1016/j.patcog.2006.12.019

[ref126] Zhang M.-L. Zhou Z.-H. (2014). A review on multi-label learning algorithms. IEEE Trans. Knowl. Data Eng. 26, 1819–1837. doi: 10.1109/TKDE.2013.39

[ref127] Zhu H. Xu G. Zhang K. Kong X. Han R. Zhou J.. (2016). Crystal structure of tyrosine decarboxylase and identification of key residues involved in conformational swing and substrate binding. Sci. Rep. 6:27779. doi: 10.1038/srep27779, PMID: 27292129 PMC4904194

[ref128] Zimmermann M. Zimmermann-Kogadeeva M. Wegmann R. Goodman A. L. (2019). Mapping human microbiome drug metabolism by gut bacteria and their genes. Nature 570, 462–467. doi: 10.1038/s41586-019-1291-3, PMID: 31158845 PMC6597290

